# Newly Identified Transcriptomic Biomarkers and Gene Signature of Pathological Complete Response to Induction Chemoimmunotherapy in Locally Advanced Head and Neck Squamous Cell Carcinoma

**DOI:** 10.1002/mco2.70582

**Published:** 2026-01-14

**Authors:** Jian‐Guo Zhou, Markus Eckstein, Haitao Wang, Tianjun Lan, Benjamin Frey, Xin Li, Xiaofan Lu, Gunther Klautke, Thomas Illmer, Maximilian Fleischmann, Simon Laban, Matthias G. Hautmann, Bálint Tamaskovics, Thomas B. Brunner, Arndt Hartmann, Rainer Fietkau, Hu Ma, Antoniu‐Oreste Gostian, Heinrich Iro, Markus Hecht, Udo S. Gaipl

**Affiliations:** ^1^ Department of Oncology The Second Affiliated Hospital of Zunyi Medical University Zunyi China; ^2^ Translational Radiobiology, Department of Radiation Oncology, Universitätsklinikum Erlangen Friedrich‐Alexander‐Universität Erlangen‐Nürnberg Erlangen Germany; ^3^ Comprehensive Cancer Center Erlangen‐EMN Erlangen Germany; ^4^ FAU Profile Center Immunomedicine (FAU I‐MED) Friedrich‐Alexander‐ Universität Erlangen‐NüRnberg Erlangen Germany; ^5^ Bavarian Cancer Research Center (Bayerisches Krebsforschungszentrum, BZKF) Erlangen Germany; ^6^ Institute of Pathology, Universitätsklinikum Erlangen Friedrich‐Alexander‐Universität Erlangen‐Nürnberg Erlangen Germany; ^7^ Center For Precision Medicine Research and Training, Faculty of Health Sciences University of Macau Macau SAR China; ^8^ Department of Oral and Maxillofacial Surgery Sun Yat‐sen Memorial Hospital of Sun Yat‐Sen University Guangzhou China; ^9^ Guangdong Provincial Key Laboratory of Malignant Tumor Epigenetics and Gene Regulation, Guangdong‐Hong Kong Joint Laboratory For RNA Medicine, Medical Research Center, Sun Yat‐Sen Memorial Hospital Sun Yat‐sen University Guangzhou China; ^10^ Department of Cancer and Functional Genomics Institute of Genetics and Molecular and Cellular Biology, CNRS/INSERM/UNISTRA Illkirch France; ^11^ Department of Radiation Oncology Hospital Chemnitz Chemnitz Germany; ^12^ Private Praxis Oncology Arnoldstraße Dresden Germany; ^13^ Department of Radiation Oncology University Hospital Frankfurt, Goethe‐Universität Frankfurt Frankfurt am Main Germany; ^14^ Department of Otolaryngology ‐ Head & Neck Surgery University Hospital Ulm Universität Ulm Ulm Germany; ^15^ Department of Radiotherapy University Hospital Regensburg Regensburg Germany; ^16^ Department of Radiotherapy and Radiation Oncology Hospital Traunstein Traunstein Germany; ^17^ Department of Radiation Oncology, Medical Faculty and University Hospital Düsseldorf Heinrich Heine University Düsseldorf Germany; ^18^ Department of Radiation Oncology Medical University of Graz Graz Austria; ^19^ Department of Radiation Oncology University Hospitals Magdeburg Magdeburg Germany; ^20^ Department of Radiation Oncology, Universitätsklinikum Erlangen Friedrich‐Alexander‐Universität Erlangen‐Nürnberg Erlangen Germany; ^21^ Department of Otolaryngology ‐ Head & Neck Surgery, Universitätsklinikum Erlangen Friedrich‐Alexander‐Universität Erlangen‐Nürnberg Elangen Germany; ^22^ Department of Otolaryngology ‐ Head & Neck Surgery Hospital Straubing Straubing Germany; ^23^ Department of Radiotherapy and Radiation Oncology Saarland University Medical Center Homburg/Saar Germany

**Keywords:** head and neck squamous cell carcinoma, immunotherapy, induction therapy, pathological complete response, transcriptomic biomarkers

## Abstract

Neoadjuvant therapies incorporating immune checkpoint inhibitors (ICIs) have shown promise in locally advanced head and neck squamous cell carcinoma (HNSCC). However, biomarkers for pathological complete response (pCR) remain undefined. In the CheckRad‐CD8 trial (NCT03426657), we performed RNA sequencing on pre‐ and post‐treatment biopsies from 77 locally advanced HNSCC patients treated with induction chemoimmunotherapy. Of these, 42 patients achieved pCR, while 35 had residual disease (RD). Differentially expressed genes (DEGs) and pathways were identified using DESeq2 and gene set enrichment analysis. Tumor immune microenvironment analysis, utilizing eight RNAseq deconvolution methods, assessed 266 gene signatures and 38 curated immunotherapy signatures. Baseline intratumoral CD8+ T‐cell density, stromal tumor lymphocyte infiltration, and combined PD‐L1 proportion score were associated with pCR. Pretreatment analysis identified 830 DEGs between pCR and RD, with T and B‐cell‐related pathways enriched in pCR samples. Logistic regression models indicated the significance of T and B cells and IFN‐gamma signaling in predicting pCR. Furthermore, a new CheckRad‐7‐gene signature, with an AUC of 0.902 in the training cohort, effectively predicted pCR and survival, serving as a robust biomarker for prognosis and treatment response in HNSCC.

## Graphical abstract

Scheme and pathological characteristics of the CheckRad‐CD8 trial cohort used for the transcriptome analyses. (A) Flow diagram depicting the patient cohort and study scheme as the basis for whole transcriptome profiling of samples from locally advanced HNSCC patients treated with induction chemoimmunotherapy (iCIT). Patients with pCR or increased CD8+ T cells entered radioimmunotherapy (RIT), patients with stable or decreased CD8+ T cells received chemoradiation or salvage surgery; (B) boxplot of intratumoral CD8+ cell density for samples from the three pathologic response groups. Intratumoral CD8+ cell density of samples from patients with pCR, PPR, and NPR was compared using the Wilcoxon rank‐sum test; (C) boxplot of sTIL for samples from the three pathologic response groups. sTIL of samples from patients with pCR, PPR, and NPR were compared using the Wilcoxon rank‐sum test; (D) boxplot of TPS for samples from the three pathologic response groups. TPS of samples from patients with pCR, PPR and NPR were compared using the Wilcoxon rank‐sum test; (E) boxplot of CPS for samples from the three pathologic response groups. CPS of samples from patients with pCR, PPR, and NPR were compared using the Wilcoxon rank‐sum test.

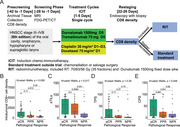

## 1 Introduction

Head and neck squamous cell carcinomas (HNSCCs) rank among the most prevalent malignancies globally with around two‐thirds of HNSCC patients presenting with a locally advanced disease stage at initial diagnosis [1]. While induction chemotherapy regimens (iCT) have demonstrated high response rates in locally advanced HNSCC, subsequent surgery or radiotherapy has not significantly improved overall survival (OS) [2]. In the past years, anti‐PD‐1 targeting immune checkpoint inhibitors (ICI) have entered first‐line therapy settings for recurrent and/or metastatic (R/M) HNSCC. ICIs have been shown to induce or reinvigorate antitumoral antigen‐specific immune reactions including various immune cells such as cytotoxic T cells, T helper cells, macrophages, and dendritic cells [3]. By blocking the interaction between PD‐1 on immune cells and PD‐L1 on tumor cells, these inhibitors enhance the immune system's ability to recognize and attack tumor cells, which may lead to durable responses in a subset of patients.

The favorable results of ICI for R/M HNSCC led to a significant interest in ICI activity and efficacy in curative settings, that is, for neoadjuvant or induction therapy regimens [4]. In other entities such as non‐small cell lung cancer (NSCLC) and melanoma, neoadjuvant immunotherapy with or without chemotherapy followed by surgery significantly improved pathological response and recurrence‐free survival in the curative therapy setting [5] [6]. Similarly, previous studies demonstrated pathologically confirmed objective response rates at the primary tumor site of 8‒35% after neoadjuvant ICI in locally advanced HNSCC [7, 8, 9, 10, 11, 12]. Induction chemoimmunotherapy (iCIT) is similar to neoadjuvant chemoimmunotherapy, but currently is mainly applied for larynx preservation or in patients with unresectable locally advanced non‐metastasized HNSCC followed by radiation treatment with curative intent. Systematic data, however, on the performance of iCIT and the influence on the tumor immune microenvironment (TIME) are scarce.

Thus, we performed the CheckRad‐CD8 investigator‐initiated phase II clinical trial (NCT03426657), where a single cycle of iCIT (combination of cisplatin/docetaxel with programmed cell death ligand 1 [PD‐L1] and cytotoxic T lymphocyte‐associated antigen 4 [CTLA‐4] inhibitors durvalumab plus tremelimumab) led to pathologically confirmed complete response rates (pCR) of 48% and improved progression‐free survival (PFS) in patients with locally advanced HNSCC [13, 14]. Moreover, a comparative retrospective analysis of single‐cycle induction chemotherapy with or without immunotherapy indicated a higher efficacy of induction chemotherapy when combined with immunotherapy [15]. Despite response rate and outcome improvements through the combination of chemotherapy with ICI, the mechanistic background of iCIT's benefits is poorly understood, highlighting the need to further substantiate modes of action and to identify reliable biomarkers for predicting favorable responses and outcomes under iCIT. In clinical practice, PD‐L1 expression remains the only widely used biomarker in HNSCC, which, however, does not allow robust identification of patients with durable ICI responses. Already performed translational research suggests many factors determining resistance or response to ICI besides PD‐L1, including architecture of the TIME, interferon gamma (IFN‐gamma) signaling, hypoxia, transforming growth factor‐beta (TGF‐β), and many other pathways [8, 12, 16, 17].

However, previously reported cohorts only contain small sample sizes, while our dataset comprises matched pre‐ and post‐treatment tissues of 35 patients who underwent a systematic treatment with iCIT. For RNAseq analyses, 33 paired samples were available. To further substantiate current knowledge on TIME factors driving responses or resistance to iCIT in our unique cohort of patients with matched pre‐ and post‐treatment tissue samples, we performed systematic pathological assessment, quantification of key immune cell populations, PD‐L1 assessment, and transcriptome‐wide mRNA sequencing (RNAseq) to identify pathological patterns and transcriptional signatures driving therapy responses or resistance in locally advanced HNSCC patients undergoing iCIT.

## 2 Results

### 2.1 Baseline Intratumoral Cytotoxic CD8+ T‐Cell Infiltration, Stromal Tumor Lymphocyte Infiltration, and Combined PD‐L1 Score are Associated With Pathological Response Patterns

**FIGURE 1:**
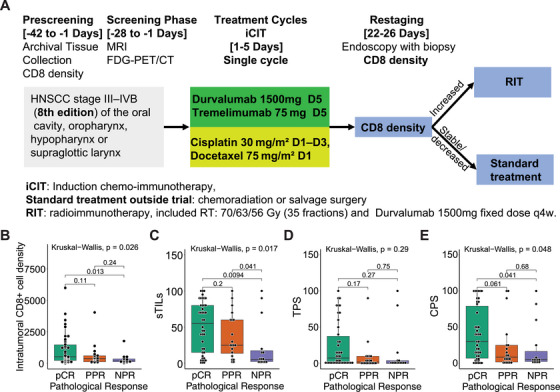
Scheme and pathological characteristics of the CheckRad‐CD8 trial cohort used for the transcriptome analyses. (A) Flow diagram depicting patient cohort and study scheme as basis for whole transcriptome profiling of samples from locally advanced HNSCC patients treated with induction chemoimmunotherapy (iCIT). Patients with pCR or increased CD8+ T cells entered radioimmunotherapy (RIT), patients with stable or decreased CD8+ T cells received chemoradiation or salvage surgery; (B) boxplot of intratumoral CD8+ cell density for samples from the three pathologic response groups. Intratumoral CD8+ cell density of samples from patients with pCR (*n* = 42), PPR (*n* = 20), and NPR (*n* = 15) was compared using the Wilcoxon rank‐sum test; (C) boxplot of sTIL for samples from the three pathologic response groups. sTIL of samples from patients with pCR (*n* = 42), PPR (*n* = 20), and NPR (*n* = 15) were compared using the Wilcoxon rank‐sum test; (D) boxplot of TPS for samples from the three pathologic response groups. TPS of samples from patients with pCR (*n* = 42), PPR (*n* = 20), and NPR (*n* = 15) were compared using the Wilcoxon rank‐sum test; (E) boxplot of CPS for samples from the three pathologic response groups. CPS of samples from patients with pCR (*n* = 42), PPR (*n* = 20), and NPR (*n* = 15) were compared using the Wilcoxon rank‐sum test.

Eighty patients treated with the iCIT combination of durvalumab plus tremelimumab and cisplatin plus docetaxel were enrolled in the phase II CheckRad‐CD8 trial (NCT03426657) across eight German centers between February 28, 2017, and October 25, 2019 (Figure 1A). The data cut‐off was January 17, 2021. mRNA sequencing was performed on 77 baseline primary tumor biopsies and 35 post‐iCIT tumor‐containing re‐biopsy samples, which were paired with 35 of the baseline primary tumor samples. A pCR was observed in 42 patients (54.5%), while 35 patients (45.5%) had residual disease (RD). Paired baseline and re‐biopsy primary tumor samples were available for RNAseq analysis for 33 RD patients. Twenty RD patients achieved a partial pathologic response (PPR) defined by substantial regressive tumor changes, scarring, and cytopathic tumor cell changes, and 13 patients exhibited a nonpathologic response (NPR) defined by tumor cells without any regressive and cytopathic changes. Detailed information on clinical efficacy has been previously reported [13, 14], and the clinicopathological features of all samples and paired samples are displayed in Tables 1 and 2, respectively.

**TABLE 1:** Clinical and pathological features of locally advanced HNSCC patients treated with induction chemoimmunotherapy.

	pCR (*n* = 42)	RD (*n* = 35)	*p*
**Sex**			0.5
Male	36	27	
Female	6	8	
**ECOG PS**			0.488
0	35	26	
1	7	9	
**Primary tumor site**			0.009
Oral cavity	5	4	
Oropharynx	24	18	
Hypopharynx	8	6	
Larynx	5	7	
**T category**			0.19
T1	3	2	
T2	10	2	
T3	11	5	
T4	18	26	
**N category**			0.201
N0	11	9	
N1	6	11	
N2	16	12	
N3	9	3	
**Tobacco smoking status**			0.004
Current smoker	18	14	
Former smoker	13	19	
Never smoker	11	2	
**HPV p16 status**			0.524
Negative	26	25	
Positive	16	10	
**TPS**			0.327
<1%	17	19	
≥1%	25	16	
**CPS**			0.5
<1%	6	8	
≥1%	36	27	
**sTIL**			0.2
<10%	10	14	
≥10%	32	21	

**TABLE 2:** Clinical and pathological features of paired HNSCC patients treated with induction chemoimmunotherapy.

	PPR (*n* = 20)	NPR (*n* = 13)	*p*
**Sex**			0.431
Male	14	11	
Female	6	2	
**ECOG PS**			0.903
0	15	10	
1	5	3	
**Primary tumor site**			0.793
Oral cavity	3	1	
Oropharynx	11	6	
Hypopharynx	2	3	
Larynx	4	3	
**T category**			0.561
T1	1	1	
T2	2	0	
T3	4	1	
T4	13	11	
**N category**			0.359
N0	4	4	
N1	9	2	
N2	5	6	
N3	2	1	
**Tobacco smoking status**			0.257
Current smoker	6	7	
Former smoker	12	6	
Never smoker	2	0	
**HPV p16 status**			0.264
Negative	13	11	
Positive	7	2	
**TPS**			0.480
<1%	9	8	
≥1%	12	5	
**CPS**			0.261
<1%	3	0	
≥1%	17	13	
**sTIL**			0.065
<10%	4	7	
≥10%	16	6	

We found that baseline intratumoral CD8^+^ cytotoxic T‐cell density (Kruskal–Wallis *p *= 0.026; Figure 1B), stromal tumor lymphocyte infiltration (sTILs) (Kruskal–Wallis *p *= 0.017; Figure 1C) and combined PD‐L1 proportion score (CPS; Kruskal–Wallis *p *= 0.048; Figure 1E) were correlated with pathological response patterns, while no correlation could be found between PD‐L1 tumor proportion score (TPS) and pathologic response patterns (*p *= 0.29; Figure 1D). Specifically, intratumoral CD8+ cytotoxic T‐cell densities and CPS were significantly higher in patients who achieved a pCR compared with those with NPR. However, no significant difference was observed between PPR patients and either NPR or pCR patients. The sTILs were significantly higher in both pCR and PPR patients compared with NPR patients, but no significant difference was found between pCR and PPR patients. TPS showed no significant differences among the three groups (data available in Table S1).

### 2.2 Gene Expression Pathways Mainly Related to Adaptive Immunity and Epithelial to Mesenchymal Transition Correlate With Pathological Response Patterns

**FIGURE 2:**
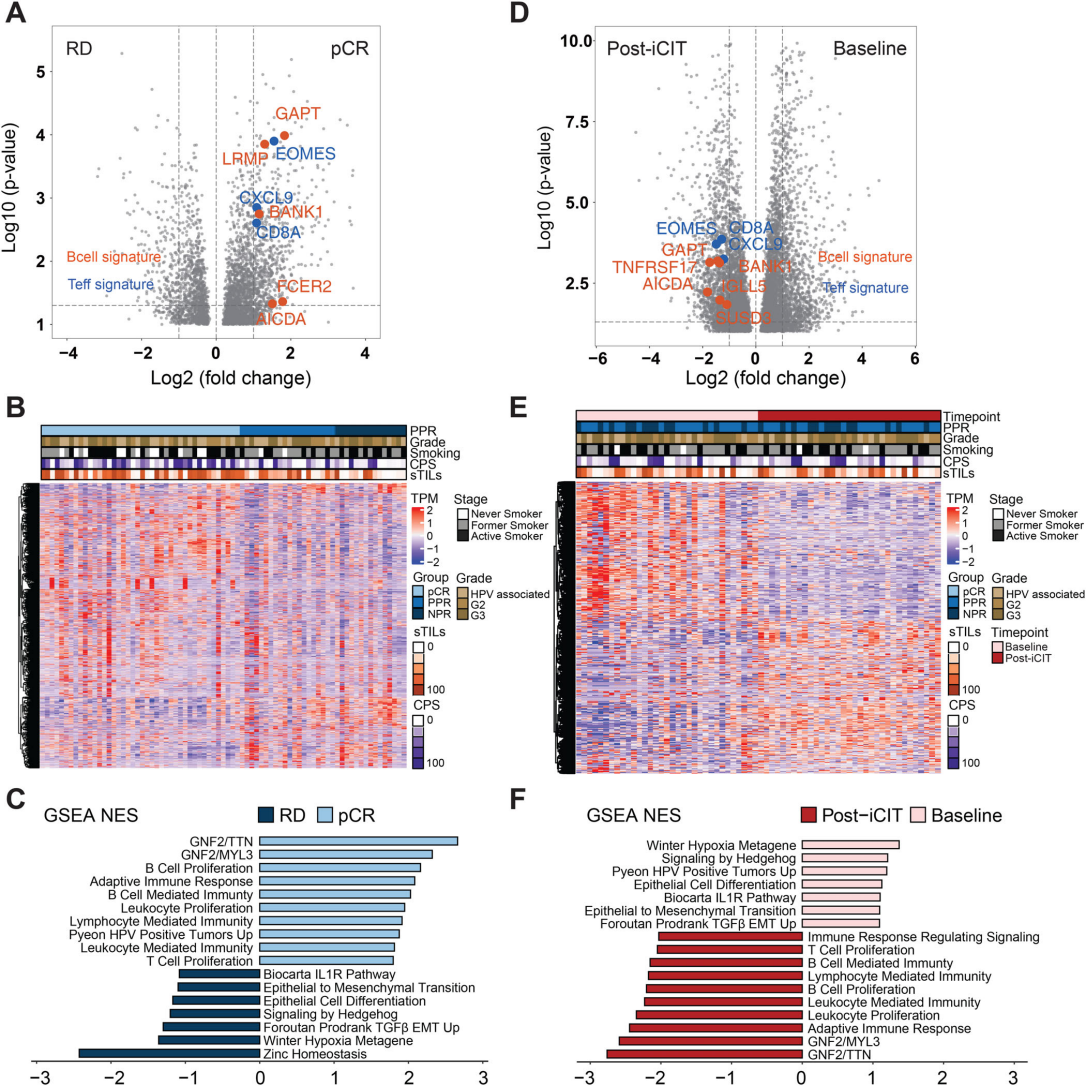
Transcriptomic dynamics in pathological response patterns in locally advanced HNSCC patients treated with iCIT. (A) Volcano plot depicting differentially expressed genes (FDR *p* < 0.05; absolute logFC ≥ 1) between iCIT‐treated HNSCC patients with pCR (*n* = 35) versus RD (*n* = 33) at baseline. Hallmark B cell genes and T‐effector genes are represented in green and red, respectively; (B) hierarchical clustering of the three pathological response patterns identified from transcriptome analyses; (C) GSEA for selected gene lists of differentially expressed genes (DEGs) in two groups (pCR vs. RD); (D) Volcano plot depicting differentially expressed genes (FDR *p* < 0.05; absolute logFC ≥ 1) between iCIT‐treated HNSCC patients with RD at baseline (*n* = 33) versus at post‐iCIT (*n* = 33). Hallmark B cell genes and T‐effector genes are represented in green and red, respectively; (E) Hierarchical clustering of the time points identified from transcriptome analyses; (F) GSEA for selected gene lists of differentially expressed genes (DEGs) in two groups (baseline vs. post‐iCIT).

We next analyzed genes and pathways (Table S2) associated with pathological responses by conducting differential gene expression and gene set enrichment analysis (GSEA) of pre‐ and post‐treatment samples. In the pre‐treatment stage, we identified 830 differentialy expression genes (DEGs) comprising 619 upregulated and 211 downregulated genes between pCR and RD patterns (Figure 2A,B and Table S3). Furthermore, we found 989, 1438, and 888 DEGs when comparing pCR versus PPR, pCR versus NPR, and PPR versus NPR, respectively (Figure S1).

In the post‐iCIT stage, 530 DEGs distinguished PPR from NPR patterns. Between pre‐ and post‐iCIT, we discovered 2314, 2500, and 1836 DEGs in RD (Figure 2D,E), PPR, and NPR patients, respectively (Figure S2 and Table S4).

To better understand the biological significance of the discovered DEGs, we performed GSEAs. When comparing pre‐treatment samples of patients with pCR versus those with RD, a significant enrichment in pathways associated with effective antitumoral immune responses was revealed. Besides kinase inhibitors, immune cells of the adaptive immune system (T and B cells) were prominent. Conversely, a negative correlation was, for example, observed with hypoxia, TGF‐β, and epithelial‐related events, such as epithelial to mesenchymal transition (EMT) (Figure 2C and Table S5). Similar findings were obtained for pCR versus PPR, pCR versus NPR, and PPR versus NPR (Figure S1 and Tables S6–S8). Immune‐associated pathways—including T cell proliferation, B cell proliferation, and adaptive immune response—were more prominently enriched in patients achieving pCR, indicating a more robust immune activation and potentially enhanced therapeutic efficacy in this group. In contrast, patients classified as non‐pathological responders (NPR) demonstrated enrichment of pathways related to EMT and epithelial cell differentiation, suggesting an increased metastatic capacity of the tumors. The partial pathological response (PPR) group exhibited an intermediate profile, lacking significant enrichment in either immune‐related pathways or those associated with aggressive tumor phenotypes when compared with pCR and NPR groups.

However, in the post‐iCIT stage, the 33 patients who did not achieve pCR also showed significant enrichment in adaptive immune cell proliferation and immunity, suggesting a reversal of immune suppression (Figure 2F and Table S9). Furthermore, PPR tumors showed upregulated TGF‐β signaling and EMT, which both play crucial roles in the remodeling of the stroma. This might indicate that these tumors are undergoing partial scarring or experience ongoing resorptive inflammation (Figure S2C and Table S10). In contrast, in the NPR group, no substantial changes regarding these parameters were observed (Figure S2F and Table S11). No significant pathways associated with pCR were observed in comparison with PPR and NPR at post‐iCIT timepoint (Figure S2I and Table S12).

Comparative enrichment analyses—both between patients achieving pCR and those with RD, as well as pre‐ versus post‐treatment—revealed that the principal functional pathways associated with the gene expression signatures clustered into two overarching categories: tumor‐intrinsic signals and immune‐related signals. Among tumor‐intrinsic pathways, those related to cellular stemness were significantly enriched, suggesting that stem‐like properties of tumor cells may influence responsiveness to ICI therapy (iCIT). EMT signaling was notably more pronounced in the RD group, particularly in NPRs, consistent with the established role of EMT in mediating therapeutic resistance. Regarding immune‐related pathways, the pCR group showed marked enrichment in adaptive immune responses—including T and B cell activation and interferon‐gamma (IFN‐γ) signaling—indicating that a pre‐existing, active immune microenvironment is likely critical for achieving complete tumor regression. In contrast, tumors from RD patients displayed elevated hypoxia and TGF‐β signaling, both of which are implicated in immune evasion and resistance to immunotherapy.

It must be emphasized that in baseline samples, more EMT is ongoing (Figure 2F). Since tumors with RD also exhibited elevated EMT signaling, too (Figure 2C), EMT appears to be involved in mediating therapy resistance.

We further repeated the DGE and GSEA analyses using tumor purity as a covariate. The results showed that the gene enrichment patterns related to the immune microenvironment and adaptive pathways remained consistent with our previous findings. Immune‐related signaling pathways were significantly enriched in pCR patients and post‐treatment patients, while intrinsic tumor signaling pathways were notably enriched in NPR patients and at baseline (Figures S3–S5).

Additionally, we performed correlation and bootstrap analyses on the outputs of different deconvolution methods to assess the consistency and uncertainty of the results. The analysis revealed that immune cells such as CD8+ T cells and B cells, which showed significant differences across the three patient groups, exhibited a moderate to high correlation across different methods, with statistical significance (Figure S6). This indicates strong concordance among the results of the various tools used. The uncertainty analysis further indicated that the standard errors for these methods were relatively consistent across the three patient groups, suggesting that the results are stable and reliable (Figures S7–S9).

### 2.3 Logistic Regression Models Point Toward a Prominent Role of the Adaptive Immune System and IFN‐Gamma in Predicting pCR

**FIGURE 3:**
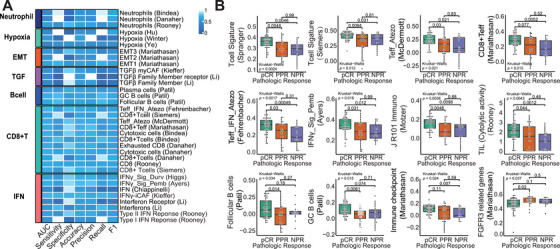
Diagnostic performance and association of gene expression signatures and TIME subtypes for pCR in HNSCC patients treated with iCIT. (A) Diagnostic performance of gene expression signatures for predicting pCR in HNSCC patients treated with iCIT; (B) boxplot of gene expression signatures for samples from the three pathologic response groups. Each gene expression signature of samples from patients with pCR, PPR, and NPR was compared using the Wilcoxon rank‐sum test.

We then applied logistic regression models predicting pCR to evaluate the connection between molecular gene signatures and pathological response in locally advanced HNSCC patients undergoing iCIT (Figure 3A and Table S13).

We found several immune‐related gene signatures, derived from five prior studies [18, 19, 20, 21, 22] that showed enriched expression in the pCR group. Again, cells of the adaptive immune system (T and B cells) and IFN‐gamma were prominent. Those signatures included, for example, the CD8+Teff signature, IMmotion150 T effector signature, 10‐gene IFNγ signature, 13‐gene inflammatory signature, T‐cell signature, Teff_IFN_Atezo (Fehrenbacher), and J R101 Immuno (Motzer) (Figure 3B).

To investigate the impact of elevated IFN‐γ activity on immune checkpoint proteins and antigen processing and presentation mechanisms (APM), we selected the APM gene set from Molecular Signatures Database (MSigDB), including APM gene sets, MHC‐I, and MHC‐II‐related APM gene sets, for single‐sample GSEA (ssGSEA) analysis to assess APM activation. Additionally, we selected CPS, TPS, and CD274 as indicators of PD‐L1 expression. By comparing the pCR, MPR, and NPR groups, we found that both APM pathway activation and PD‐L1 expression were significantly higher in the pCR group compared with the NPR group (Figure S10). These results are consistent with the IFN‐γ enrichment trends we previously predicted, suggesting that elevated IFN‐γ activity can activate the APM pathway and enhance PD‐L1 expression.

However, these signatures did not differ between the PPR and NPR groups, most likely suggesting that PPR is an effect of chemotherapy, while these tumors did not respond adequately to the immunotherapy component of the iCIT regimen. We then assessed the capacity of the significantly enriched signatures to predict pCR in HNSCC patients treated with iCIT and revealed a good predictive performance in our cohort with reaching a minimum area under the curve (AUC) of 0.68.

To further validate the predictive specificity of these immune signatures, we evaluated 266 relevant molecular gene signatures from the IOBR package [23]. The CD8+T cell and CD8+Teff‐related gene signatures yielded similar AUC levels (0.64–0.70). The gene signatures related to IFN‐γ, TGF‐β, EMT, and hypoxia may not exhibit better AUC performance compared with gene signatures associated with T cells and CD8+ effector T cells (Figure 3A and Table S13).

### 2.4 Dynamic Changes of TIME Factors, Angiogenic Signatures, and DNA Repair Factors Contribute to PPR in RD

**FIGURE 4:**
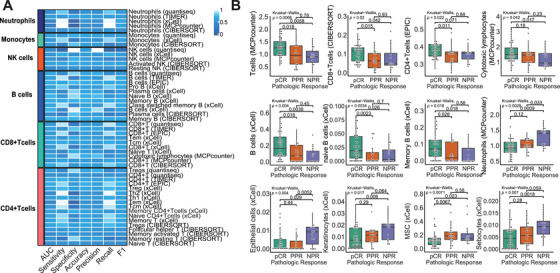
Diagnostic performance and association of TIME subtypes for pCR in HNSCC patients treated with iCIT. (A) Diagnosis performance of TIME subtypes for predicting pCR in HNSCC patients treated with iCIT; (B) boxplot of TIME subtypes for samples from the three pathologic response groups. Each TIME subtype of samples from patients with pCR, PPR, and NPR was compared using the Wilcoxon rank‐sum test.

We then systematically investigated the relationship between the TIME and therapy response patterns (Figure 4A and Table S14). The logistic regression models indicated positive correlations of pCR with lymphoid, myeloid, and stromal cells, including CD8^+^/cytotoxic T‐cells, CD4^+^ T‐cells, naive B‐cells, and memory B cells. Tumors not responding to iCIT (NPR) showed decreased levels of T cells, but increased keratinocyte and epithelial cell content. Enrichment with mesenchymal stem cells (MSCs), neutrophil granulocytes, and sebocytes was present (Figure 4B). These observations underscore the significance of EMT and extracellular matrix (ECM) remodeling in such tumors, which sheds light on the potential mechanisms related to resistance to iCIT. Activated stroma remodeling takes place in nonresponding tumors. To follow up on these observations, we correlated the pathological scoring of CD8+ T cells and stroma content assessment with calculated tumor purity and stromal immune cell admixture and found positive correlations (Figure S11; tumor purity: *R* = 0.51, *p *< 0.001; stroma purity vs. estimate *R* = 0.44, *p *< 0.001; stroma purity vs. xCell *R* = 0.33, *p *= 0.004).

Corroborating the above gene expression and pathological findings, we found that tumors with subsequent pCR were enriched for ImmuneScore, Microenvironment Score, ESTIMATE Score, and Immunophenoscore for effector cells (EC_IPS) score (Figure S12). On the other hand, tumors with subsequent RD after iCIT (PPR or NPR) were depleted for these scores and showed significantly higher TumorPurity scores, indicating immune depletion compared with tumor cell amounts. However, here no significant differences were observed in StromalScore (Figure S13). The predictive performance of these TIME cell types or estimates for pCR was variable with AUCs ranging between 0.29 and 0.72, while T cells, CD8^+^ T cells, CD4^+^ T cells, naïve B cells, and memory B cells demonstrated the highest predictive accuracy with all estimates yielding AUCs of 0.68 or higher (Figure 4A).

**FIGURE 5:**
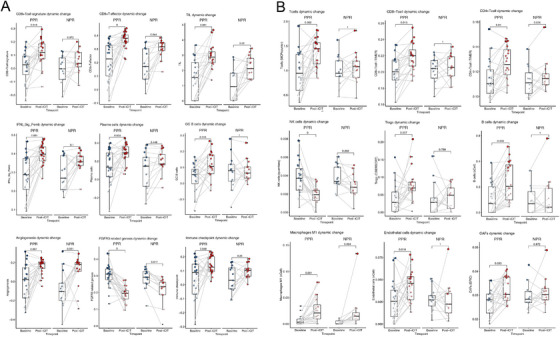
Transcriptional signature and TIME dynamic change of response patterns between pre‐ and post‐treatment. (A) Expression levels of transcriptional signatures in pre‐ and post‐treatment samples of the PPR (*n* = 20) and NPR (*n* = 13) groups of patients. Statistical comparisons were performed using the paired Wilcoxon signed‐rank test; (B) abundances of TIME subtypes in pre‐ and post‐treatment samples of PPR (*n* = 20) and NPR (*n* = 13) groups of patients. Statistical comparisons were performed using the paired Wilcoxon signed‐rank test.

To characterize the dynamic transcriptional signature and TIME landscape of different response patterns, we performed paired comparisons between baseline and post‐treatment in 33 patients with RD (Figures 5 and S14). This group included 20 patients with PPR and 13 patients with NPR. In this context, it is important to stress that PPR tumors showed clear cytopathological changes, tumor regression, and an increase in immune infiltration, while NPR tumors were unaffected when compared with matched baseline tumor samples.

Comparison of transcriptional signature profiles of PPR tumors before and after iCIT mirrored these pathological changes. Specifically, we observed an increased infiltration of cells of the adaptive immune system contributing to effective antitumor responses, such as several T effector signatures (CD8+ and CD4+ T cells, T effector cells, increased antigen‐specific plasma cell differentiation), upregulation of immune checkpoints, and B cell infiltration (Figures 5A and S14A). Additionally, cells of the innate immune system (NK cells) and cells bridging innate and adaptive immunity (DCs, macrophages) were increased. Moreover, fibroblast growth factor receptor 3 and angiogenic signatures were significantly increased, reflecting neo‐angiogenesis that fuels the influx of immune cells. Furthermore, stroma remodeling and M1 macrophage infiltration were increased, reflecting resorptive inflammation and tissue regeneration after tumor cell killing (endothelial cells, CAFs, M1 macrophages, mast cells; Figures 5B and S14B). Interestingly, gene signatures including DNA damage repair, DNA replication, nucleotide excision repair, and cell cycle activity usually stemming from rapidly replicating cells, for example, tumor cells, were significantly decreased in pre/post comparisons of PPR tumors. This might reflect the mitotic arrest and damage to the tumor cells (Figures 5B and S14B). In contrast, tumors not showing any pathological response (NPR) did not show increased influx of immune cells, alterations of DNA damage and related events or signals for increased angiogenesis or resorptive inflammation. This reflects a strong therapy resistance (details provided in Table S15).

One has further to stress here that neutrophils (deconvoluted with quanTiseq) significantly decreased in PPR after iCIT, and conversely did not significantly change in NPR. This suggests that neutrophils might serve as a secondary barrier against T‐cell attacks on cancer cells, potentially contributing to cancer immunotherapy resistance. Releasing this secondary barrier could be crucial in enhancing the response rates, particularly in cancers abundant in neutrophils [24].

### 2.5 Immune Stem Cells, CD8 T Cells, B Cells, and Adaptation Pathways Correlate With Pathological Response Patterns

To validate the bioinformatics analysis, we performed multiplex immunofluorescence (mIHC) staining of formalin‐fixed paraffin‐embedded (FFPE) tumor samples from 12 patients with HNSCC. Several key markers were assessed: HIF‐1α and GLUT1 to evaluate tumor metabolic activity and hypoxic adaptation, TGF‐β to reflect tumor response to hypoxic stress, and immune cell markers (CD8a for cytotoxic T cells, CD11c for dendritic cells, CD20 for B cells, and TCF7 for immune stem cells) to assess immune infiltration. Additionally, markers such as CD31 and pan‐CK were used to investigate endothelial cell activity and EMT in the tumor microenvironment.

**FIGURE 6:**
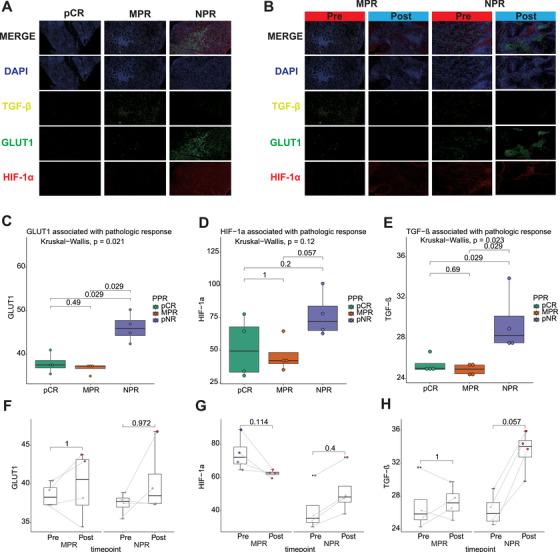
Correlation of adaptation pathways in pathological response patterns and between pre‐ and post‐treatment. (A and B) The confocal microscopy images show the coexpression of HIF‐1α, GLUT1, TGF‐β in each pathological response pattern and treatment stage by immunofluorescence analysis. DAPI: blue, TGF‐β: yellow, GLUT1: green, HIF‐1α: red. Scale bar, 100 µm. Boxplots illustrating the association of GLUT1 (C), HIF‐1α (D), and TGF‐β (E) with pathologic response in different treatment groups. Statistical significance is determined by Kruskal–Wallis tests, and the *p* values are indicated. Boxplots showing the changes in expression of GLUT1 (F), HIF‐1α (G), and TGF‐β (H) from pretreatment to post‐treatment in the MPR and NPR groups. Paired statistical analysis is performed, with *p* values shown for each comparison.

mIHC staining revealed that pCR patients had significantly lower expression of these markers compared with NPR patients, suggesting a reduced metabolic activity and a diminished ability of tumor cells to adapt to hypoxic conditions. In contrast, MPR patients displayed intermediate expression levels of HIF‐1α, GLUT1, and TGF‐β, which corresponded to a partial response to treatment (Figure 6A,B). Specifically, for HIF‐1α (*p* = 0.029) and TGF‐β (*p* = 0.029), significant differences were found between pCR and NPR patients (Figure 6C–E). Moreover, we noticed that the expression of these markers remained relatively stable in MPR patients post‐treatment, but in NPR patients, there was a marked increase in HIF‐1α, GLUT1, and TGF‐β levels after treatment, indicating tumor progression and treatment resistance. While the expression showed no significant difference (*p* = 0.057), the data trend is still noteworthy and suggests that tumor metabolic suppression is associated with a favorable therapeutic response (Figure 6F–H).

To explore the mechanisms by which HIF‐1α influences response, we conducted correlation analyses between HIF1A expression and gene signature scores related to DNA damage repair, DNA replication, and cell cycle activity. The results revealed that HIF1A expression did not show a significant correlation with DNA damage repair. However, statistically significant correlations were observed with both DNA replication and cell cycle activity (Figure S15). While HIF1A showed a weak correlation with DNA replication (*R* = 0.32) and cell cycle activity (*R* = 0.21), trends observed in the quantification of mIHC staining provide some insight. These findings suggest that in HNSCC, HIF1A may not exert its effects through these pathways.

**FIGURE 7:**
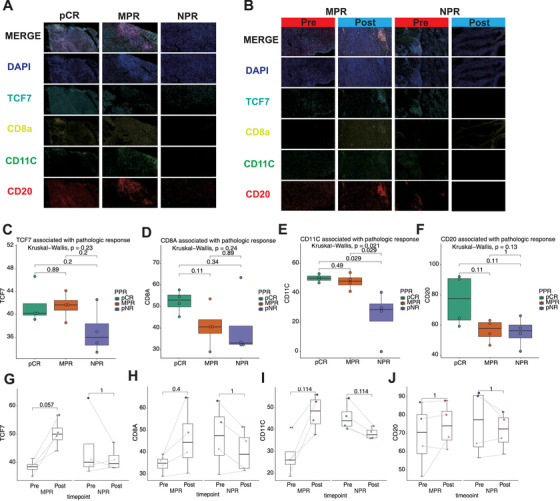
Correlation of the tumor immune microenvironment in pathological response patterns and between pre‐ and post‐treatment. (A and B) The confocal microscopy images show the coexpression of TCF7, CD11c, CD20, CD8a in each pathological response pattern and treatment stage by immunofluorescence analysis. DAPI: blue, TCF7: cyan, CD8a: yellow, CD11C: green, CD20: red. Scale bar, 100 µm. Boxplots illustrating the association of TCF7 (C), CD11C (D), CD20 (E), and CD8a (F) with pathologic response in different treatment groups. Statistical significance is determined by Kruskal–Wallis tests, and the *p* values are indicated. Boxplots showing the changes in expression of TCF7 (G), CD11C (H), CD20 (I), and CD8a (J) from pretreatment to post‐treatment in the MPR and NPR groups. Paired statistical analysis is performed, with *p* values shown for each comparison.

Regarding immune cell infiltration, pCR patients exhibited the highest levels of immune cell infiltration, particularly CD8a, CD11c, and CD20. This suggests a robust immune response in pCR patients, which could enhance treatment efficacy and tumor control (Figure 7A,B). Analysis of CD8a expression confirmed the differential trends between the groups, supporting the notion that higher cytotoxic T cell infiltration is associated with a better response to treatment (Figure 7C–F). In contrast, NPR patients demonstrated limited immune cell infiltration, with relatively low expression of immune markers, which could indicate an inadequate or ineffective immune response. MPR patients showed an intermediate pattern of immune cell infiltration, with slight increases in immune cells post‐treatment, suggesting a moderate immune response. While markers like CD11c and CD20 did not reach statistical significance, the trends still reflect differences in immune cell presence across the groups (Figure 7G–J).

For EMT, we found that in NPR patients, post‐treatment tumor tissue exhibited a decrease in pan‐CK expression, suggesting a shift toward a mesenchymal‐like phenotype, which is commonly associated with tumor progression and metastasis (Figure S16). This EMT‐like transition was not observed in MPR patients, whose pan‐CK expression remained stable, indicating that their tumors likely maintained an epithelial phenotype and exhibited less aggressive behavior. CD31 expression, a marker of endothelial cells and tumor angiogenesis, was increased in NPR patients post‐treatment, suggesting that tumor angiogenesis may play a role in the progression of treatment‐resistant tumors. Conversely, MPR patients exhibited decreased CD31 expression, reflecting a reduction in tumor vasculature and possibly indicating tumor growth inhibition. These findings suggest that EMT and endothelial activity play key roles in treatment resistance and tumor progression.

### 2.6 Development and Validation of a Novel Gene Signature for Predicting pCR in HNSCC Following iCIT

Although we found promising immune‐ and EMT‐related patterns of response or resistance to iCIT in HNSCC, the prediction of pCR remains challenging. We proceeded to identify an applicable classifier to predict patients potentially benefitting from iCIT. Thus, we identified and validated a novel gene signature using LASSO regression within our CheckRad cohort. Genes linked to our described observations were included: CARD10, which can be recruited and activated by T and B cell receptors; CD4; CD6, which facilitates adhesion between T cells and antigen‐presenting cells; leukocyte‐associated immunoglobulin‐like receptor‐1 (LAIR1), which is expressed on T cells, B cells, macrophages, and dendritic cells; Toll‐like receptor 10 (TLR10) which elicit anti‐inflammatory effects; TRIM72 that quickly senses the change of oxygen partial pressure; and RYR1 being connected to neuromuscular disorders but has also been discussedas playing a role in dendritic cell increased surface expression of the major histocompatibility complex. The signature formula is as follows: score = −0.83829116*CARD10 + 0.46187363*CD4 + 1.23201267*CD6 + 0.37466309*LAIR1 − 0.09622538* + RYR1 + 0.20856138*TLR10 + 1.08842602*TRIM72.

To validate the reliability of the model, we performed the Hosmer–Lemeshow (HL) test and calibration slope analysis for the seven‐signature model. The HL test yielded a *p* value of 0.086, indicating an acceptable fit between the model's predictions and the observed outcomes. The calibration slope was 0.9416, which is very close to 1, demonstrating excellent calibration and alignment with the actual data. Furthermore, the mean absolute error (MAE) was 0.063 and the mean squared error was 0.00527, confirming the model's robust predictive accuracy. To further assess the clinical utility of the model, we conducted decision curve analysis, which indicated that the 7‐gene signature provided superior clinical benefit to patients, particularly across a range of decision thresholds (Figure S17).

This novel identified gene signature showed the best performance for discriminating pCR from RD in training and test cohorts (Figure 7A,B). High expression of the gene signature is strongly associated with pathological response (two‐tailed *t*‐test, *p* = 1.53e−07), with the association driven by the pCR group (pCR vs. PPR, *p* = 1.91e−07; pCR vs. NPR, *p* = 5.38e−05; PPR vs. NPR, *p* = 0.75; Figure 7C).

Next, we conducted analysis on the ChecKRad‐7 gene signature and model scores in PPR and NPR paired samples. The results showed that there were significant changes in the expression of five genes in PPR patients pre‐ and posttreatment, while only two genes in NPR patients showed significant changes in expression. Although there was a significant increase in model scores for both groups of patients after treatment, there was a significant difference in overall model scores between PPR patients and NPR patients (*p* = 0.018; Figure S18).

To assess whether the model's classification performance improves, we compared its predictive efficacy with the commonly used clinical indicators, CPS and sTILs, for distinguishing patients who respond to treatment. The results showed that the 7‐gene signature outperformed both CPS (AUC = 0.662) and sTILs (AUC = 0.655), achieving superior predictive efficacy (AUC = 0.871; Figure S19).

To further investigate and validate the prognostic role of the identified CheckRad‐7‐gene signature for HNSCC patients, we collected published transcriptome datasets and included The Cancer Genome Atlas (TCGA) with 486 HNSCC patients, the Johns Hopkins cohort with 47 HPV‐positive oropharyngeal cancer patients (GSE112026), and the Hutch cohort with 97 HPV‐negative oral squamous cell carcinoma patients (GSE41613).

**FIGURE 8:**
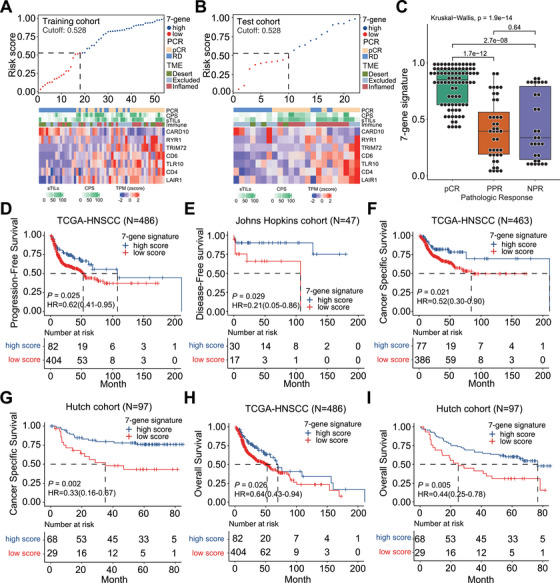
Identification and validation of the CheckRad‐7‐gene signature for predicting pCR and prognosis. (A) The risk score and CheckRad‐7‐gene heatmap of each HNSCC patient (patient ID) treated with iCIT in the training cohort; (B) The risk score and CheckRad‐7‐gene heatmap of each HNSCC patient (patient ID) treated with iCIT in the test cohort; (C) boxplot of CheckRad‐7‐gene signature for all samples from the three pathologic response groups. The CheckRad‐7‐gene signature of samples from patients with pCR, PPR, and NPR were compared using Wilcoxon rank‐sum test; (D) Kaplan–Meier curve comparing PFS between patients with high and low CheckRad‐7‐gene signature scores in TCGA‐HNSCC (*N* = 486) (HR_PFS_ = 0.62(0.41–0.95), *p* = 0.025, Log‐rank test); (E) Kaplan–Meier curve comparing DFS between patients with high and low CheckRad‐7‐gene signature score in Johns Hopkins cohort (*N* = 47) (HR_DFS_ = 0.21(0.05–0.86), *p* = 0.029, Log‐rank test); (F) Kaplan–Meier curve comparing CSS between patients with high‐ and low CheckRad‐7‐gene signature score in TCGA‐HNSCC (*N* = 463) (HR_CSS_ = 0.52(0.30–0.90), *p* = 0.021, Log‐rank test); (G) Kaplan–Meier curve comparing CSS between patients with high and low CheckRad‐7‐gene signature score in Hutch cohort (*N* = 97) (HR_CSS_ = 0.33(0.16–0.67), *p* = 0.002, Log‐rank test); (H) Kaplan–Meier curve comparing OS between patients with high‐ and low CheckRad‐7‐gene signature score in TCGA‐HNSCC (*N* = 486) (HR_OS_ = 0.64(0.43–0.94), *p* = 0.026, Log‐rank test); (I) Kaplan–Meier curve comparing OS between patients with high‐ and low CheckRad‐7‐gene signature score in Hutch cohort (*N* = 97) (HR_OS_ = 0.44(0.25–0.78), *p* = 0.005, Log‐rank test). Hazard ratio (HR) and confidence intervals (CIs) based on stratified Cox models are shown along with log‐rank *p* values, and statistical tests were two‐sided.

We demonstrated that patients with a high CheckRad‐7‐gene signature reach longer PFS (*p* = 0.025; Figure 8D). Similar results were obtained for disease‐free survival (DFS) (*p* = 0.029; Figure 8E), cancer‐specific survival (CSS) (Figure 8G; *p* = 0.021 and *p* = 0.002, respectively), and OS (Figure 8H and I; *p* = 0.026 and *p* = 0.005, respectively). The CheckRad‐7‐gene signature not only predicts pathological response, but also demonstrates prognostic power.

Subsequently, we performed an independent prognostic validation of the CheckRad‐7‐gene signature using the TCGA HNSCC dataset, comprising data of *N* = 486 HNSCC patients. In order to include known risk factors such as HPV status, surgical margin status, lymphovascular invasion, and perineural invasion, we further filtered the TCGA patient data through cBioPortal [25], selecting 31 patients who had complete data for all variables to perform multivariable analysis. The findings demonstrated that the CheckRad‐7 gene signature was significantly associated with patient prognosis, irrespective of whether OS, PFS, or CSS was used as the clinical outcome measurement. This association remained significant in multivariable Cox regression analysis after adjusting for potential confounding variables (P_OS = 0.022, P_CSS = 0.034, P_PFS = 0.034; see Table S16). Additionally, we performed both univariate and multivariate logistic regression analyses on the CheckRad‐CD8 cohort to validate the independence of our signature. We included factors that are known to significantly influence tumor response, such as p16 status, tumor stroma, ECOG performance status, baseline CPS, and sTILs. The results showed that our model score remains an independent prognostic factor (p_univariate = 0.001, p_multivariate = 0.013). In summary, the CheckRad‐7 gene signature appears to function as an independent prognostic marker for patients with HNSCC.

## 3 Discussion

In this study, we examined associations between baseline and posttreatment transcriptomic biomarkers and pathological response to a single cycle of induction with cisplatin and docetaxel combined with tremelimumab and durvalumab in locally advanced HNSCC patients and assessed dynamic changes of the TIME after induction chemoimmunotherapy and, finally, idnetified the complementary CheckRad‐7‐gene signature for HNSCC patients.

To our knowledge, this study offers the largest cohort of locally advanced HNSCC patients with tissue samples from baseline and after iCIT. We found 830 DEGs between pCR and RD patterns, with the pCR group being enriched in pathways associated with effective adaptive antitumoral immune responses. Further, in baseline samples, more EMT is ongoing, and tumors with RD showed a higher EMT signaling, too. The pCR group therefore shows a “hot” tumor microenvironment.However, patients with RD were enriched in and correlated with hypoxia, but showed less infiltration of immune cells contributing to antitumor immunity (“cold” tumor). We, for example, found BPIFB2 being highly expressed in NPR group, which can be the reason for decreased CD8+T cells attraction, and thereby contributing to immune resistance [26]. However, high expression of SMC1B in the PPR group potentially drives somatic chromosome instability, which is responsible for the response to immunotherapy [27].

Conversely, no significant pre‐treatment‐associated pathways were identified between PPR and NPR at the post‐iCIT. We would like to clarify that, although no significant pCR‐related pathways were observed when comparing post‐PPR and post‐NPR groups, this does not diminish the importance of the dynamic immune and molecular changes observed within the PPR group. The absence of significant differences between baseline PPR versus NPR and post‐PPR versus post‐NPR does not contradict the within‐group changes (baseline vs. post) observed in the PPR group, but not in the NPR group. Instead, it highlights that key molecular and tumor/immune reprogramming events occur dynamically over time in PPR tumors, but not in NPR tumors, which explains why post‐PPR and post‐NPR profiles appear similar despite the clear treatment‐induced changes in the PPR group. This underscores the value of multi‐timepoint (pre‐ and pos‐ttreatment) dynamic analysis and suggests that further deconvolution and higher‐resolution profiling could help identify specific signaling pathways that drive PPR, but fail to induce changes in NPR. Moreover, epithelial dedifferentiation, which was enriched in the NPR group, may indicate immune suppression that contributes to the ineffectiveness of iCIT against cancer [28].

Indeed, among RD cases, there was a significant difference in TIME immune subsets in pre‐ and post‐iCIT samples. Dynamic changes of immune‐related factors, angiogenic signatures, and DNA repair factors contribute to PPR in RD. This highlights the interplay between DNA damage and repair factors and immune alterations in the tumor and its microenvironment [29].

The DEGs of patients that show PPR between pre‐ and post‐treatment include T lymphocyte differentiation and proliferation, suggesting a transfer to an immune‐enriched tumor microenvironment. In cases with NPR, we also observed a high number of DEGs (206/502) in the residual tissues, even including some of the similar differentiation and proliferation signatures of immune subsets as in the PPR group. However, tumors not showing any pathological response did not show alterations of DNA damage and related events, signals for increased angiogenesis, or increased influx of immune cells.

With the current detailed analyses, we confirmed our previous observations that particularly pretreatment CD8^+^ T cells are significantly associated with a higher pCR rate in HNSCC [14, 30]. We now systematically screened the dynamic TIME landscape and transcriptional signature of different response patterns between baseline and post‐iCIT.

Our analyses revealed a contribution of EMT and ECM in tumors not responding to iCIT. However, dynamic changes of TIME factors, angiogenic signatures and DNA repair factors contributed to PPR in RD after iCIT. While T effector cells, B cells, NK cells, and dendritic cells increased, neutrophils significantly decreased. Hints exist that neutrophil extracellular traps can stimulate EMT [31]. Our analyses suggest that in PPR after iCIT, low neutrophils and less EMT might contribute to response rates. Recent publications have highlighted concerns regarding the application of EMT‐related signatures in samples with variable tumor cell content. To further validate our analytical findings, we performed mIHC [32]. TGF‐β–induced EMT represents a critical step in tumor invasion and metastasis. Previous studies have demonstrated that TGF‐β facilitates EMT progression through activation of the PI3K–PKB–mTOR signaling pathway [33]. Therefore, we employed TGF‐β expression as a surrogate marker for EMT activity. Our results revealed significantly lower TGF‐β expression in tumor tissues from patients achieving pCR compared with NPR, suggesting reduced EMT activity in the pCR group. In contrast, elevated TGF‐β expression in NPR patients indicated activation of the EMT pathway, consistent with our previous analyses.

Further, logistic regression models to predict pCR indicated a prominent role of T and B cells and IFN‐gamma signaling. Several immunotherapy survival benefit predictive gene signatures were already identified that showed significantly high expression in the pCR group of CD8+Teff signature [18, 20], IFNγ signature [34], and 26‐gene JAVELIN Renal 101 Immuno signature [22]. However, these gene signatures predicted pCR with intermediate performance regarding AUC values.

Thus, we trained a diagnostic model based on DEGs for improvement of predictive power. We thereby succeeded to identify and validate a novel CheckRad‐7‐gene signature including CARD10, CD4, CD6, LAIR1, RYR1, TLR10, and TRIM72, which predicts pCR with higher performance compared with other published signatures in the CheckRad cohort.

All of these seven genes had significantly higher baseline expression, while CARD10 and Ryanodine receptor 1(RYR1) had significantly lower baseline expression in the patients, who subsequently experienced pCR. These results are consistent with in vitro experiments and clinical studies examining CARD10 and indicating inhibition of tumor growth, especially after NF‐κB inactivation [35]. Moreover, RYR1 mutational status correlates with TMB as panel variable for survival prediction in surgically treated patients with oral cavity squamous cell carcinoma [36].

In our analyses, CD4+ T cells were higher expressed in patients with pCR than with RD. It has become obvious that some CD4+ helper T (TH) cell subsets, such as TH1 and TH9, support the cytotoxicity of CD8+ T cells and NK cells and activate antitumor immunity [37]. The previous finding that CD6 is expressed by T‐lymphocytes and NK cells to increase cancer cell death [38] supports our newly identified gene signature. Furthermore, the high expression of LAIR‐1 in the pCR group indicates that LAIR‐1 drives apoptotic signaling and proliferation of CD8+ T and Treg cells to enhance the efficacy of antitumor immunotherapy [39, 40]. It has also been shown that higher TLR10 expression was significantly positively correlated with higher infiltrating levels of B cells, which contributed to the response to ICI treatment [41, 42]. The high expression of tripartite motif containing 72 (TRIM72) in patients with pCR is consistent with findings from lung cancer, where it was shown to inhibit cellular proliferation and tumor progression, and to enhance sensitivity to cisplatin [43].

Our analysis demonstrated that in all patients treated with iCIT, regardless of pathological response, most of immune subsets increased. Exceptions were resting and activated memory CD4+ T cells, epithelial cells, and MSCs. Taken together, iCIT might turn “immune cold” cancers into “immune hot” tumors [7, 44].

Others have shown that hypoxia is inversely related to pathological response in HNSCC patients treated with neoadjuvant immunotherapy [8, 12]. In agreement, we found a negative correlation with hypoxia, TGF‐beta, and EMT when comparing pretreatment samples of patients with pCR versus those with RD. However, we did not find a difference between the PPR and NPR group, most probably due to the small sample size. However, one might also speculate that PPR may be mostly due to that the tumors responded well to chemotherapy, but not to immunotherapy. This might explain the lack of immunological differences in these cohorts.

Antiangiogenic therapy (e.g., angiogenesis inhibitors or anti‐VEGF/VEGFR2 monoclonal antibodies, such as anlotinib, bevacizumab, ramucizumab, and lenvatinib) combined with immune‐chemotherapy or immunotherapy might convert some patients from RD to pCR [45, 46]. These might be mechanisms of therapy resistance to iCIT. We also identified that CD8+ T cell‐related gene signature and deconvolution including memory CD8+ T cell [47] and CD8+TIL signature [16] had a strong ability to predict pCR to iCIT, which indicates that therapies fostering tumor infiltration of lymphocytes alone or combined with ICI may prolong survival in patients with initially lower expression of TILs [48].

There are some limitations of this study. Diagnosis and pCR evaluation were based on tissue biopsy in the CheckRad‐CD8 study. However, most neoadjuvant immunotherapy studies evaluated pathologic response according to tumor viability in the surgical specimen after neoadjuvant immunotherapy. Because of our chosen biopsy‐based approach, there was not enough tissue for whole exome sequencing, DNA methylation array, and BCR. Further, our analysis is based on bulk RNAseq to identify the correlation of gene expression, TIME abundances, and pathologic response. Liu et al. demonstrated that FLT4 mutation enrichment favors response, but CDKN2A, YAP1, or JAK2 mutations correlated with resistance [7]. Furthermore, they found that promoter methylation contributes to immunotherapy resistance [49]. Recent studies have also indicated that genomics‐based analysis of tumor heterogeneity is limited. Combining spatial transcriptome selection analysis is a strategy for future elucidation of heterogeneity [50]. Another concern is that this study did not perform lymph node biopsy on patients after treatment. Although some studies have confirmed that imaging methods can predict the status of lymph node metastasis and pathological response, lymph node biopsy is still the main method to determine pathological response [51]. In the future, multi‐omics landscape analyses including clinical, peripheral blood [30], digital pathology [52], genomic, and transcriptomic profiles that all do represent the tumor ecosystem and are candidates as predictors for cancer therapy response [53] should therefore performed in a complementary manner.

## 4 Conclusions

Our analyses revealed that immune‐associated transcriptomic features, dynamic changes in the TIME, but also EMT to be associated with pCR to iCIT in locally advanced HNSCC. The parameters identified thereby are functionally connected to a novel CheckRad‐7‐gene signature as a potent biomarker for predicting pCR after iCIT, but also for prognosis in selected patient cohorts. One should additionally keep in mind that patients treated with iCIT treatment turned “immune cold” cancers into “immune hot” tumors, regardless of the pathological response. This emphasizes that, besides immune alterations (particularly those of the adaptive immune system), EMT and ECM are key factors for predicting pCR in HNSCC.

## 5 Method

### 5.1 Ethics

This study provides exploratory biomarker analyses for CheckRad‐CD8 trial (ClinicalTrials.gov Identifier: NCT03426657) which was approved by the Institutional Review Board of the Friedrich‐Alexander‐Universität Erlangen‐Nürnberg (No.131_18 Az). All patients gave written informed consent before the first study procedures were performed. Tissue was obtained with written, informed patient consent. All relevant ethical regulations were correctly followed, and samples were fully anonymized.

### 5.2 Patients and Treatment

Eligible patients had histologically confirmed HNSCC stage III‐IVB of the oral cavity, oropharynx, hypopharynx, or supraglottic larynx. iCIT treatment consisted of a single cycle of induction chemo‐immunotherapy with cisplatin 30 mg/m^2^ body surface area (BSA) on Days 1–3 and docetaxel 75 mg/m^2^ BSA on Day 1. Tremelimumab (anti‐CTLA4) fixed dose of 75 mg and durvalumab (anti‐PDL1) fixed dose of 1500 mg were both administered on Day 5. Restaging assessment consisted of diagnostic imaging and endoscopy including representative re‐biopsy of the primary tumor area was performed on Day 22–26 (Figure 1A).

### 5.3 Patient Tissue Collection

Biopsies were obtained by an experienced otolaryngologic oncologist based on either the visible residual tumor or the originally documented and imaged tumor area, then immediately FFPE.

### 5.4 Immunohistochemistry

PD‐L1 (closed SP263 assay), CD8 (C8/144B, mouse monoclonal) immunohistochemistry of FFPE primary tumor samples was performed on a BenchMark Ultra autostainer (Ventana Medical Systems) as described previously [13, 14]. CPS is a method used to assess the expression of PD‐L1 on both tumor cells and immune cells. It is calculated by dividing the number of PD‐L1 positive cells by the total number of tumor cells, then multiplying by 100 to get the percentage. Stromal tumor infiltrating lymphocytes (sTILs) refer to lymphocytes infiltrating the tumor's stroma. This is quantified by assessing the percentage of the stroma area occupied by lymphocytes under a microscope. STIL levels were measured according to current guidelines of the International TILs Working Group; in brief, the amount of desmoplastic tumor stroma occupied by mononuclear immune cells (excluding granulocytes) was assessed semiquantitatively (0–100%) by excluding necrotic or organized necrotic areas (large fibrotic areas) from scoring [54]. Detail methods have been described in our previous report [14].

### 5.5 Pathological Response Evaluation

Pathologic response was determined on H&E‐stained, FFPE sections in the Institute of Pathology Erlangen. Patients were classified in response categories as outlined in our study proposal^14^: patients without residual invasive disease on pathological evaluation of the re‐biopsy specimen was defined as pCR. Patients with both ≤ 50% residual viable tumor cells and 50‒99% decrease in viable tumor cell percentage from baseline to postinduction chemoimmunotherapy had a pathological partial response, and patients with any percentage of residual viable tumor cells but <50% change in viable tumor cell percentage had no pathological response (NPR). The endpoint of this biomarker analysis was pCR and secondary endpoints were positive predictive value and negative predictive value.

### 5.6 Whole Transcriptome Sequencing

Total RNA was extracted from FFPE samples using a SPLIT RNA Extraction Kit (Lexogen) according to the manufacturer's instructions [55]. The QuantSeq libraries were prepared using QuantSeq 3’mRNA‐Seq Library Prep Kit for Illumina (FWD) (Lexogen), according to the manufacturer's instructions [56]. The QuantSeq libraries were sequenced on Illumina Novaseq 600 to produce 75 bp single‐end reads for each sample. Library preparation and sequencing was done at Lexogen.

The QuantSeq reads were trimmed to remove the adapter contamination, polyA read through, and low‐quality tails using BBMap with Lexogen's recommendation [57]. The trimmed reads (fastq files) were aligned to the reference genome (UCSC hg38 with annotations from GRCh38.p13) using STAR (v.2.7.1a) [58], and assembly annotated by GENCODE (gencode.v34.annotation.gtf). Gene expression was subsequently quantified using featureCounts (v. 2.0.1) [59] convert RNAseq data from count to transcripts per million (TPM). Normalized TPM matrix using the *normalize.quantiles* function in the *preprocessCore* package (v1.56.0). Expression was calculated as log2(1 + TPM).

### 5.7 Differential Gene Expression Analysis

The input data for differential gene expression analysis were read counts from gene expression level analysis. *DESeq2* package (v.1.34.0) was used to compare the differential expression between the different groups using the raw counts [60]. Data were normalized by a negative binomial distribution statistical method. The resulting *p* values were subjected to multiple test corrections according to the Benjamini and Hochberg methods to exclude false positives. The DEGs were identified when adjusted *p* value < 0.05 and |log2(Fold Change) | > 1.

### 5.8 Immune Gene Signatures Calculation

We collected 266 relevant gene molecular signatures from *IOBR* package which have been organized into three distinct categories: TME‐associated, tumor‐metabolism, and tumor‐intrinsic signatures, and 38 molecular signatures manually curated from clinical trials (Table S2) to estimate the potential predictive role for pathological response. Excepted the TIL (cytolytic activity), all of the signatures were calculated by ssGSEA [61, 62]. For each gene signature, patients were divided into two groups based on the median gene signature score of all samples: high gene signature score was defined as score at or above median levels, and low gene signature score was defined as score below the median.

### 5.9 Immune Cell Infiltration Estimation

We employed IOBR [62] for TIME decomposition seamlessly integrates eight widely‐used open‐source deconvolution methods, including CIBERSORT [63], ESTIMATE [64], quanTIseq [65], TIMER [66], IPS [67], MCPCounter [68], xCell [69], and EPIC [70].

### 5.10 Gene Set Enrichment Analysis

LogFCs estimated by *DESeq2* across the indicated comparisons (such as pCR vs. non‐pCR) were used to ranked genes for identifying significant regulated gene sets, then preformed GSEA with preranked mode (permutation was set as 20,000 times, minimum and maximum gene set sizes were set as 15–500) by *clusterProfiler* package (v4.2.2) [71]. Gene sets from the MSigDB (v2023.2) [72] and GO, KEGG, WikiPathways, and Reactome databases were used. Adjusted *p* < 0.05, false‐discovery rate (FDR) < 0.25, and normalized enrichment score (|NES|) > 1 were considered significant enrichment.

### 5.11 Multicolor Immunohistochemistry

Pretreatment tumor tissues were collected from 12 patients diagnosed with HNSCC, consisting of four patients who achieved pCR, four with major pathological response (MPR), and four with no pathological response (NPR) following therapy. MPR is defined as when more than 50% of the tumor tissue shows necrosis or tumor cell death. Posttreatment tumor tissues were also obtained from four MPR and four NPR patients. To assess tumor microenvironmental adaptation and immune cell infiltration, multiplex immunofluorescent staining was performed on FFPE tumor tissue sections. Tumor metabolic activity and hypoxic adaptation were evaluated using markers for HIF‐1α, GLUT1, and TGF‐β, while immune cell infiltration was assessed using CD8a (cytotoxic T cells), CD11C (dendritic cells), CD20 (B cells), and TCF7 (immune stem cells). Additionally, markers for endothelial cells (CD31) and epithelial cells (pan‐CK) were used to evaluate EMT and tumor microenvironmental changes.

The primary antibodies used for staining included HIF‐1α (Servicebio; catalog number: GB151339, 1:6000), GLUT1 (Servicebio; catalog number: GB15179, 1:10,000), TGF‐β (Servicebio; catalog number: GB113495, 1:10,000), CD8a (Servicebio; catalog number: GB12068, 1:15,000), CD11C (Servicebio; catalog number: GB115690, 1:5000), CD20 (Servicebio; catalog number: GB115721, 1:2500), TCF7 (Immunoway; catalog number: YM8473, 1:2000), CD31 (Servicebio; catalog number: GB11063‐1, 1:2000), pan‐CK (Servicebio; catalog number: GB122053, 1:2000), and α‐SMA (Servicebio; catalog number: GB121364, 1:10000).

### 5.12 Statistical Analysis

All statistical analyses were performed using R software, version 4.3.2 (2023‐10‐31) including two‐sample Mann–Whitney test, paired Wilcoxon signed‐rank test and the Wilcoxon rank‐sum test for continuous data, Fisher's exact test for categorical data, Pearson correlation for intercorrelations. Log‐rank test for Kaplan–Meier curves, and Cox proportional hazards regression for estimating the hazard ratios (HRs) and 95% confidence interval (CI).

The CheckRad‐cd8 study were randomly divided into training (*n* = 54) and validation cohort (*n* = 23). Least absolute shrinkage and selection operator (LASSO) regression analysis was performed for gene signature list selection and reduction from DEG using the *glmnet* package (version 3.0) with 20,000 times simulations [73]. This LASSO method shrinks coefficients toward zero, and eliminates unimportant terms entirely, thus reducing prediction error and minimizing overfitting, technical details of which have been described previously [74].The group of DEGs when AUC reached the biggest AUC in validation cohort and AUC of each cohort more than 0.85 was selected as the optimal biomarker and was used to develop the final prediction model.

The receiver operating characteristic curves and predictive accuracy (general cutoff of 0.5) were used to assess the model predictive performance. Heatmap illustration was created by R package “*ComplexHeatmap 2.5.5*” [75]. For all unadjusted comparisons, a two‐tailed *p* < 0.05 was considered statistically significant.

## Author Contributions

Study design and developed concept: Jian‐Guo Zhou, Udo S. Gaipl, and Markus Hecht. Data collection: Udo S. Gaipl, Hu Ma, Jian‐Guo Zhou, Tianjun Lan, Benjamin Frey, Gunther Klautke, Thomas Illmer, Maximilian Fleischmann, Simon Laban, Matthias G. Hautmann, Bálint Tamaskovics, Thomas B. Brunner, Arndt Hartmann, Rainer Fietkau, Antoniu‐Oreste Gostian, and Heinrich Iro. Data analysis: Jian‐Guo Zhou, Haitao Wang, Xiaofan Lu, and Xin Li. Study coordination: Markus Hecht, Udo S. Gaipl, and Jian‐Guo Zhou. Writing the manuscript: Haotao Wang, Jian‐Guo Zhou, Udo S. Gaipl, Hu Ma, Markus Hecht, and Xin Li. Obtained funding: Jian‐Guo Zhou and Markus Eckstein. Accessed and verified the data: Jian‐Guo Zhou. Decided to submit the manuscript: Markus Hecht, Markus Eckstein. All authors read and approved the final version of the manuscript.

## Funding

This research was partly supported by grants awarded to Jian‐Guo Zhou including the National Natural Science Foundation of China (Grant No. 82504050), Noncommunicable Chronic Diseases‐National Science and Technology Major Project (Grant No. 2023ZD0502105), MOE (Ministry of Education in China) Liberal arts and Social Sciences Foundation (Grant No. 24YJCZH462), Youth Science and Technology Elite Talent Project of Guizhou Provincial Department of Education (Grant No. QJJ‐2024‐333), Zunyi City Science and Technology Plan Project (Grant Nos. Zunshi Kehe HZ (2023) 142 and Zunshi Kehe HZ (2025) 256), Zunyi City Science and Technology Innovation Team (Grant No. Zun KCTD (2025) 63), Future Science and Technology Elite Talent Cultivation Project of Zunyi Medical University (ZYSE 2023‐02), Guizhou Province High‐Level Overseas‐Educated Talents Innovation and Entrepreneurship Selective Funding Program (Grant No. 3), and the Key Program of the Education Sciences Planning of Guizhou Province (Grant No. 2024A007). The current research was partly supported by grants awarded to Markus Eckstein including the Else Kröner‐Fresenius Foundation/EKFS grants 2020_EKEA.129 and 2023_EKES.07, the Clinician Scientist program of the IZKF and TOPeCS funding line of the IZKF (T04) of the FAU, an advanced research grant of the IZKF of the FAU Erlangen‐Nürnberg (IZKF‐FAU D41) and a Young Clinical Scientist Fellowship of the Bavarian Center for Cancer Research (BZKF; YSF‐TP01).

## Conflicts of Interest

The authors declare no relevant conflict of interest regarding this manuscript. Markus Hecht reports collaborations with Merck Serono (advisory role, speakers’ bureau, honoraria, travel expenses, research funding); MSD (advisory role, speakers’ bureau, honoraria, travel expenses, research funding); AstraZeneca (advisory role, speakers’ bureau, honoraria, travel expenses, research funding); Novartis (research funding); BMS (advisory role, honoraria, speakers’ bureau); Teva (travel expenses); Sanofi (advisory role, speakers’ bureau, honoraria). Udo S. Gaipl and Rainer Fietkau received support for presentation activities for Dr Sennewald Medizintechnik GmbH, have received support for investigator initiated clinical studies (IITs) from MSD and AstraZeneca and contributed at Advisory Boards Meetings of AstraZeneca and Bristol‐Myers Squibb. Markus Eckstein reports personal fees, honoraria and travel expenses received from Eisai, MSD, AstraZeneca, Janssen‐Cilag, Cepheid, Roche, Astellas, Diaceutics, Owkin, BMS; research grants received from AstraZeneca, Janssen‐Cilag, STRATIFYER, Cepheid, Roche, Gilead, Owkin, QUIP GmbH; advisory roles for Diaceutics, MSD, AstraZeneca, Janssen‐Cilag, GenomicHealth, Owkin, BMS. B.T. reports collaborations with BMS (advisory role, speakers’ bureau, honoraria); Merck Serono (advisory role, speakers’ bureau, honoraria, travel expenses); MSD (advisory role, speakers’ bureau, honoraria, travel expenses, research funding); Sanofi (advisory role, speakers’ bureau, honoraria). This study provides exploratory biomarker analyses for CheckRad‐CD8 trial (ClinicalTrials.gov Identifier: NCT03426657), which was approved by the Institutional Review Board of the Friedrich‐Alexander‐Universität Erlangen‐Nürnberg (No.131_18 Az). All patients gave written informed consent before the first study procedures were performed. Tissue was obtained with written, informed patient consent. All relevant ethical regulations were correctly followed, and samples were fully anonymized.

## Code Availability

Scripts for data processing and for generating figures are available at GitHub (https://github.com/JianGuoZhou3/CheckRad_RNAseq).

## Supporting information




**Supporting Figure 1**: Transcriptomic dynamics in pathological response patterns in locally advanced HNSCC patients treated with iCIT at baseline. (A) Volcano plot depicting differentially expressed genes (FDR *p* < 0.05; absolute logFC ≥ 1) between iCIT treated HNSCC patients with pCR versus PPR at baseline. Hallmark B cell genes and T‐effector genes are represented in darkgreen and darkred, respectively; (B) Hierarchical clustering of the PPR and NPR patterns identified from transcriptome analyses; (C) GSEA enrichment analysis for selected gene lists of differentially expressed genes (DEGs) in two groups (pCR vs. PPR); (D) Volcano plot depicting differentially expressed genes (FDR *p* < 0.05; absolute logFC ≥ 1) between iCIT treated HNSCC patients with pCR versus NPR at baseline. Hallmark B cell genes and T‐effector genes are represented in drakgreen and drakred, respectively; (E) Hierarchical clustering of the pCR and NPR patterns identified from transcriptome analyses; (F) GSEA enrichment analysis for selected gene lists of differentially expressed genes (DEGs) in two groups (pCR vs. NPR); (G) Volcano plot depicting differentially expressed genes (FDR *p* < 0.05; absolute logFC ≥ 1) between iCIT treated HNSCC patients with PPR versus NPR at baseline. Hallmark B cell genes and T‐effector genes are represented in drakgreen and drakred, respectively; (H) Hierarchical clustering of the PPR and NPR patterns identified from transcriptome analyses; (I) GSEA enrichment analysis for selected gene lists of differentially expressed genes (DEGs) in two groups (PPR vs. NPR).
**Supporting Figure 2**: Transcriptomic dynamics in pathological response patterns in locally advanced HNSCC patients with RD after iCIT.
**Supporting Figure 3**: Transcriptomic dynamics in pathological response patterns with tumor purity in patients.
**Supporting Figure 4**: Transcriptomic dynamics in pathological response patterns with tumor purity in patients at baseline.
**Supporting Figure 5**: Transcriptomic dynamics in pathological response patterns with tumor purity in patients with RD after iCIT
**Supporting Figure 6**: Correlation of CD8+ T cell and B cell between transcriptomic estimate.
**Supporting Figure 7**: Bootstrap analysis for CIBERSORT.
**Supporting Figure 8**: Bootstrap analysis for xCells.
**Supporting Figure 9**: Bootstrap analysis for other methods.
**Supporting Figure 10**: Immune‐checkpoint activity and antigen processing and presentation machinery associated with pathologic response patterns.
**Supporting Figure 11**: Correlation of immune infiltration between pathology and transcriptomic estimate.
**Supporting Figure 12**: Transcriptomic estimate characteristics associated with pathologic response patterns.
**Supporting Figure 13**: Transcriptomic estimate characteristics dynamic change of response patterns between pre‐ and posttreatment.
**Supporting Figure 14**: Transcriptional signature and TIME dynamic change of response patterns between pre‐ and posttreatment.
**Supporting Figure 15**: Correlation analysis between HIF1A and relevant pathwa.
**Supporting Figure 16**: Correlation of epithelial–mesenchymal transition between pre‐ and posttreatment.
**Supporting Figure 17**: Decision curve analysis for CheckRad‐7‐gene signature.
**Supporting Figure 18**: CheckRad‐7‐gene signature dynamic change of response patterns between pre‐ and posttreatment.
**Supporting Figure 19**: ROC curves for the 7‐gene signature, CPS, and sTILs.

**Supporting Table 1**: Clinical information of CheckRad RNAseq
**Supporting Table 2**: Published Gene Signatures
**Supporting Table 3**: Differentially expressed genes of multiple comparison with DESeq2 at baseline
**Supporting Table 4**: Differentially expressed genes of multiple comparison with DESeq2 in RD patients
**Supporting Table 5**: Gene set enrichment scores for MsigDB for pCR vs. RD patients at baseline
**Supporting Table 6**: Gene set enrichment scores for MsigDB for pCR vs. PPR patients at baseline
**Supporting Table 7**: Gene set enrichment scores for MsigDB for pCR vs. NPR patients at baseline
**Supporting Table 8**: Gene set enrichment scores for MsigDB for PPR vs. NPR patients at baseline
**Supporting Table 9**: Gene set enrichment scores for MsigDB for baseline vs. post‐iCIT in RD patients
**Supporting Table 10**: Gene set enrichment scores for MsigDB for baseline vs. post‐iCIT in PPR patients
**Supporting Table 11**: Gene set enrichment scores for MsigDB for baseline vs. post‐iCIT in NPR patients
**Supporting Table 12**: Gene set enrichment scores for MsigDB for PPR vs. NPR patients at post‐iCIT
**Supporting Table 13**: AUC of Pretreatment transcriptional signature and TME subtypes to predict pCR
**Supporting Table 14**: Pretreatment transcriptional signature and TME subtypes correlation with pCR
**Supporting Table 15**: Pre‐ and posttreatment transcriptional signature and TME dynamic change of response patterns

## Data Availability

For requesting RNA‐Seq raw sequencing data, processed gene expression data, clinical information, and response outcome information from CheckRad‐CD8 study, please contact the project leaders, Prof. Dr Udo Gaipl and Prof. Dr Markus Hecht with detailed proposals. Source data are provided with this paper, from which all processed and annotated data that underlie the figures, supplementary figures, and tables are available at Zenodo (https://zenodo.org/10.5281/zenodo.10493836). The sequencing data and clinical information of RNAseq derived from human samples have been deposited at the China National Center for Bioinformation (https://ngdc.cncb.ac.cn/omix) under accession number PRJCA043216.
