# Supplementary material for: Newly Identified Transcriptomic Biomarkers and Gene Signature of Pathological Complete Response to Induction Chemoimmunotherapy in Locally Advanced Head and Neck Squamous Cell Carcinoma
Source: MedComm (2020). 2026 Jan 14;7(1):e70582. doi: 10.1002/mco2.70582 (PMC12802090; doi:10.1002/mco2.70582)
Supplement: Supplementary file 1 — Supporting Figure 1: Transcriptomic dynamics in pathological response patterns in locally advanced HNSCC patients treated with iCIT at baseline. (A) Volcano plot depicting differentially expressed genes (FDR p < 0.05; absolute logFC ≥ 1) between iCIT treated HNSCC patients with pCR versus PPR at baseline. Hallmark B cell genes and T‐effector genes are represented in darkgreen and darkred, respectively; (B) Hierarchical clustering of the PPR and NPR patterns identified from transcriptome analyses; (C) GSEA enrichment analysis for selected gene lists of differentially expressed genes (DEGs) in two groups (pCR vs. PPR); (D) Volcano plot depicting differentially expressed genes (FDR p < 0.05; absolute logFC ≥ 1) between iCIT treated HNSCC patients with pCR versus NPR at baseline. Hallmark B cell genes and T‐effector genes are represented in drakgreen and drakred, respectively; (E) Hierarchical clustering of the pCR and NPR patterns identified from transcriptome analyses; (F) GSEA enrichment analysis for selected gene lists of differentially expressed genes (DEGs) in two groups (pCR vs. NPR); (G) Volcano plot depicting differentially expressed genes (FDR p < 0.05; absolute logFC ≥ 1) between iCIT treated HNSCC patients with PPR versus NPR at baseline. Hallmark B cell genes and T‐effector genes are represented in drakgreen and drakred, respectively; (H) Hierarchical clustering of the PPR and NPR patterns identified from transcriptome analyses; (I) GSEA enrichment analysis for selected gene lists of differentially expressed genes (DEGs) in two groups (PPR vs. NPR). Supporting Figure 2: Transcriptomic dynamics in pathological response patterns in locally advanced HNSCC patients with RD after iCIT. Supporting Figure 3: Transcriptomic dynamics in pathological response patterns with tumor purity in patients. Supporting Figure 4: Transcriptomic dynamics in pathological response patterns with tumor purity in patients at baseline. Supporting Figure 5: Transcriptomic dynamics in p [file MCO2-7-e70582-s002.docx]

# Newly identified transcriptomic biomarkers and gene signature of pathological complete response to induction chemoimmunotherapy in locally advanced head and neck squamous cell carcinoma

Jian-Guo Zhou ^1,2,3,4,23,24#^, Markus Eckstein^3,5,23#^, Haitao Wang^6,#,^^, Tianjun Lan^7,8#^, Benjamin Frey^2,3,4,23^,Xin Li^1,24^, Xiaofan Lu^9^, Gunther Klautke^10^, Thomas Illmer^11^, Maximilian Fleischmann^12^, Simon Laban^13^, Matthias G. Hautmann^14,15^, Bálint Tamaskovics^16^, Thomas B. Brunner^17,18^, Arndt Hartmann^3,4,5,23^, Rainer Fietkau^3,20,23^, Hu Ma^1,24^, Antoniu-Oreste Gostian^3,19,21,23^, Heinrich Iro^3,19,23^, Markus Hecht^22,^*, Udo S. Gaipl^2,3,4,23^*^+^

^1^ Department of Oncology, The Second Affiliated Hospital of Zunyi Medical University, Zunyi, 563000, P. R. China.

^2^ Translational Radiobiology, Department of Radiation Oncology, Universitätsklinikum Erlangen, Friedrich-Alexander-Universität Erlangen-Nürnberg, Erlangen, Germany

^3^ Comprehensive Cancer Center Erlangen-EMN, Erlangen

^4^ FAU Profile Center Immunomedicine (FAU I-MED), Friedrich-Alexander- Universität Erlangen-Nürnberg, Erlangen, Germany

^5^Institute of Pathology, Universitätsklinikum Erlangen, Friedrich-Alexander-Universität Erlangen-Nürnberg, Erlangen, Germany

^6^ Center for Precision Medicine Research and Training, Faculty of Health Sciences, University of Macau, Macau SAR, People's Republic of China

^7^ Department of Oral and Maxillofacial Surgery, Sun Yat-sen Memorial Hospital of Sun Yat-sen University, Guangzhou 512141, China

^8^Guangdong Provincial Key Laboratory of Malignant Tumor Epigenetics and Gene Regulation, Guangdong-Hong Kong Joint Laboratory for RNA Medicine, Medical Research Center, Sun Yat-sen Memorial Hospital, Sun Yat-sen University, Guangzhou 511131, China

^9^ Department of Cancer and Functional Genomics, Institute of Genetics and Molecular and Cellular Biology, CNRS/INSERM/UNISTRA, 68400 Illkirch, France.

^10^ Department of Radiation Oncology, Hospital Chemnitz, Chemnitz, Germany

^11^ Private Praxis Oncology, Arnoldstraße, Dresden, Germany

^12^ Department of Radiation Oncology, University Hospital Frankfurt, Goethe-Universität Frankfurt, Frankfurt am Main, Germany

^13^ Department of Otolaryngology - Head & Neck Surgery, University Hospital Ulm, Universität Ulm, Ulm, Germany

^14^ Department of Radiotherapy, University Hospital Regensburg, Regensburg, Germany

^15^ Department of Radiotherapy and Radiation Oncology, Hospital Traunstein, Traunstein, Germany

^16^ Department of Radiation Oncology, Medical Faculty and University Hospital Düsseldorf, Heinrich Heine University, Düsseldorf, Germany

^17^ Department of Radiation Oncology, Medical University of Graz, Graz, Austria

^18^ Department of Radiation Oncology, University Hospitals Magdeburg, Magdeburg, Germany

^19^ Department of Otolaryngology - Head & Neck Surgery, Universitätsklinikum Erlangen, Friedrich-Alexander-Universität Erlangen-Nürnberg, Elangen, Germany

^20^ Department of Radiation Oncology, Universitätsklinikum Erlangen, Friedrich-Alexander-Universität Erlangen-Nürnberg, Erlangen, Germany

^21^ Department of Otolaryngology - Head & Neck Surgery, Hospital Straubing, Germany

^22^ Department of Radiotherapy and Radiation Oncology, Saarland University Medical Center, Homburg/Saar, Germany

^23^ Bavarian Cancer Research Center (Bayerisches Krebsforschungszentrum, BZKF), Erlangen, Germany

^24^ Key Laboratory for Cancer Prevention and treatment of Guizhou Province, Zunyi, 563000, P. R. China

Jian-Guo Zhou, Markus Eckstein, Haitao Wang, and Tianjun Lan are joint first authors.

* Markus Hecht and Udo S. Gaipl are joint senior authors.

^^^ current address：Thoracic Surgery Branch, Center for Cancer Research, National Cancer Institute, Bethesda, Maryland

^+^ Corresponding author:

Correspondence to: Prof. Dr. Udo Gaipl; [udo.gaipl@uk-erlangen.de](mailto:udo.gaipl@uk-erlangen.de), Universitätsstr. 27, Translational Radiobiology, Department of Radiation Oncology, Universitätsklinikum Erlangen & Friedrich-Alexander-Universität Erlangen-Nürnberg (FAU), 91054, Erlangen, Germany.

**Running Title:** Transcriptomic biomarkers predict pCR to chemo-immunotherapy in head and neck squamous cell carcinoma

# sTable legends

Table S1. Clinical information of CheckRad RNAseq

Table S2. Published Gene Signatures

Table S3. Differentially expressed genes of multiple comparison with DESeq2 at baseline

Table S4. Differentially expressed genes of multiple comparison with DESeq2 in RD patients

Table S5. Gene set enrichment scores for MsigDB for pCR vs. RD patients at baseline

Table S6. Gene set enrichment scores for MsigDB for pCR vs. PPR patients at baseline

Table S7. Gene set enrichment scores for MsigDB for pCR vs. NPR patients at baseline

Table S8. Gene set enrichment scores for MsigDB for PPR vs. NPR patients at baseline

Table S9. Gene set enrichment scores for MsigDB for baseline vs. post-iCIT in RD patients

Table S10. Gene set enrichment scores for MsigDB for baseline vs. post-iCIT in PPR patients

Table S11. Gene set enrichment scores for MsigDB for baseline vs. post-iCIT in NPR patients

Table S12. Gene set enrichment scores for MsigDB for PPR vs. NPR patients at post-iCIT

Table S13. AUC of Pre-treatment transcriptional signature and TME subtypes to predict pCR

Table S14. Pre-treatment transcriptional signature and TME subtypes correlation with pCR

Table S15. Pre- and post-treatment transcriptional signature and TME dynamic change of response patterns

# sFigure legends


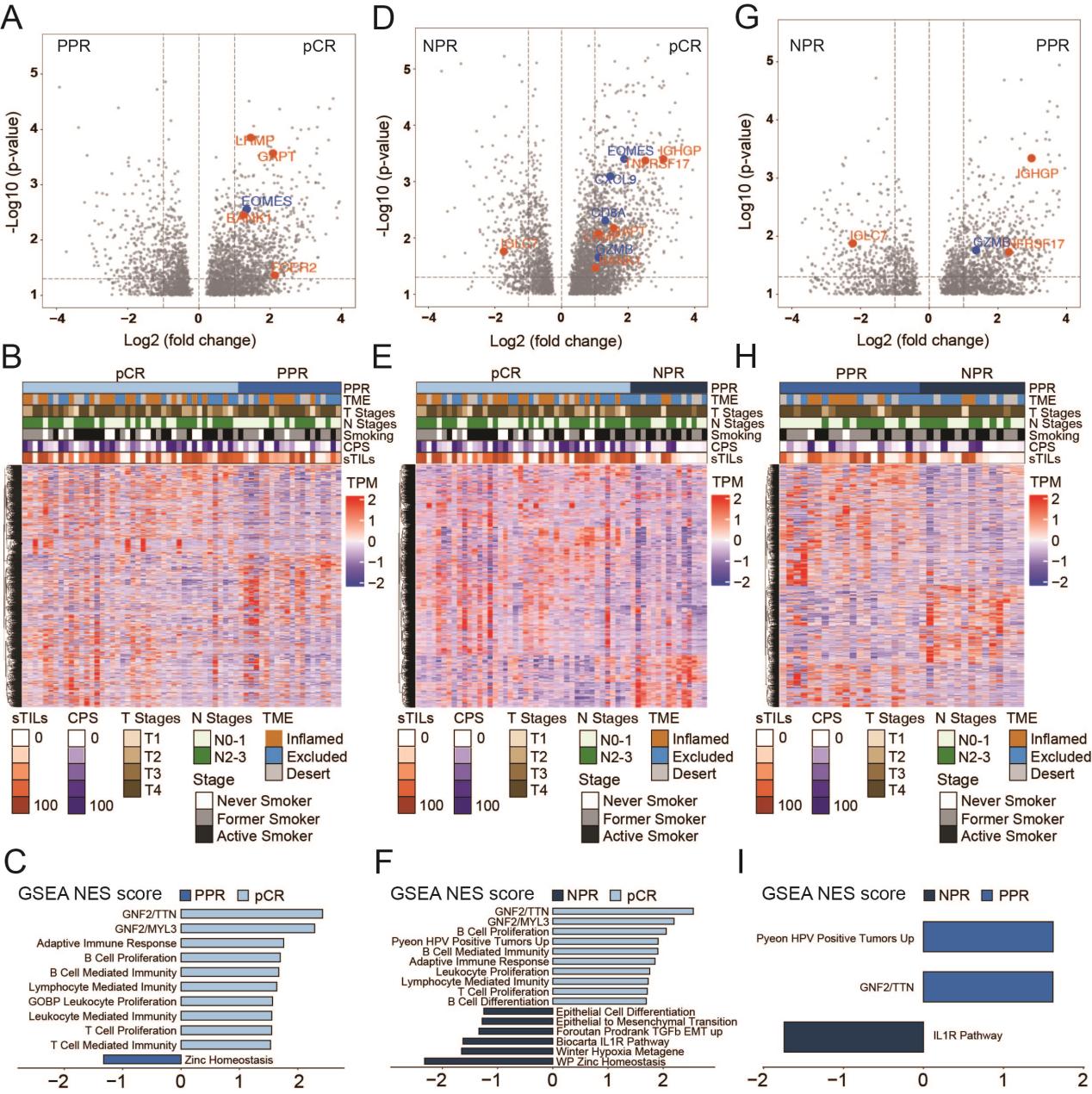


**Figure S1. Transcriptomic dynamics in pathological response patterns in locally advanced HNSCC patients treated with iCIT at baseline.**

(A) Volcano plot depicting differentially expressed genes (FDR p<0.05; absolute logFC ≥1) between iCIT treated HNSCC patients with pCR versus PPR at baseline. Hallmark B cell genes and T-effector genes are represented in darkgreen and darkred, respectively; (B) Hierarchical clustering of the PPR and NPR patterns identified from transcriptome analyses; (C) GSEA enrichment analysis for selected gene lists of differentially expressed genes (DEGs) in two groups (pCR vs. PPR); (D) Volcano plot depicting differentially expressed genes (FDR p<0.05; absolute logFC ≥1) between iCIT treated HNSCC patients with pCR versus NPR at baseline. Hallmark B cell genes and T-effector genes are represented in drakgreen and drakred, respectively; (E) Hierarchical clustering of the pCR and NPR patterns identified from transcriptome analyses; (F) GSEA enrichment analysis for selected gene lists of differentially expressed genes (DEGs) in two groups (pCR vs. NPR); (G) Volcano plot depicting differentially expressed genes (FDR p<0.05; absolute logFC ≥1) between iCIT treated HNSCC patients with PPR versus NPR at baseline. Hallmark B cell genes and T-effector genes are represented in drakgreen and drakred, respectively; (H) Hierarchical clustering of the PPR and NPR patterns identified from transcriptome analyses; (I) GSEA enrichment analysis for selected gene lists of differentially expressed genes (DEGs) in two groups (PPR vs. NPR).


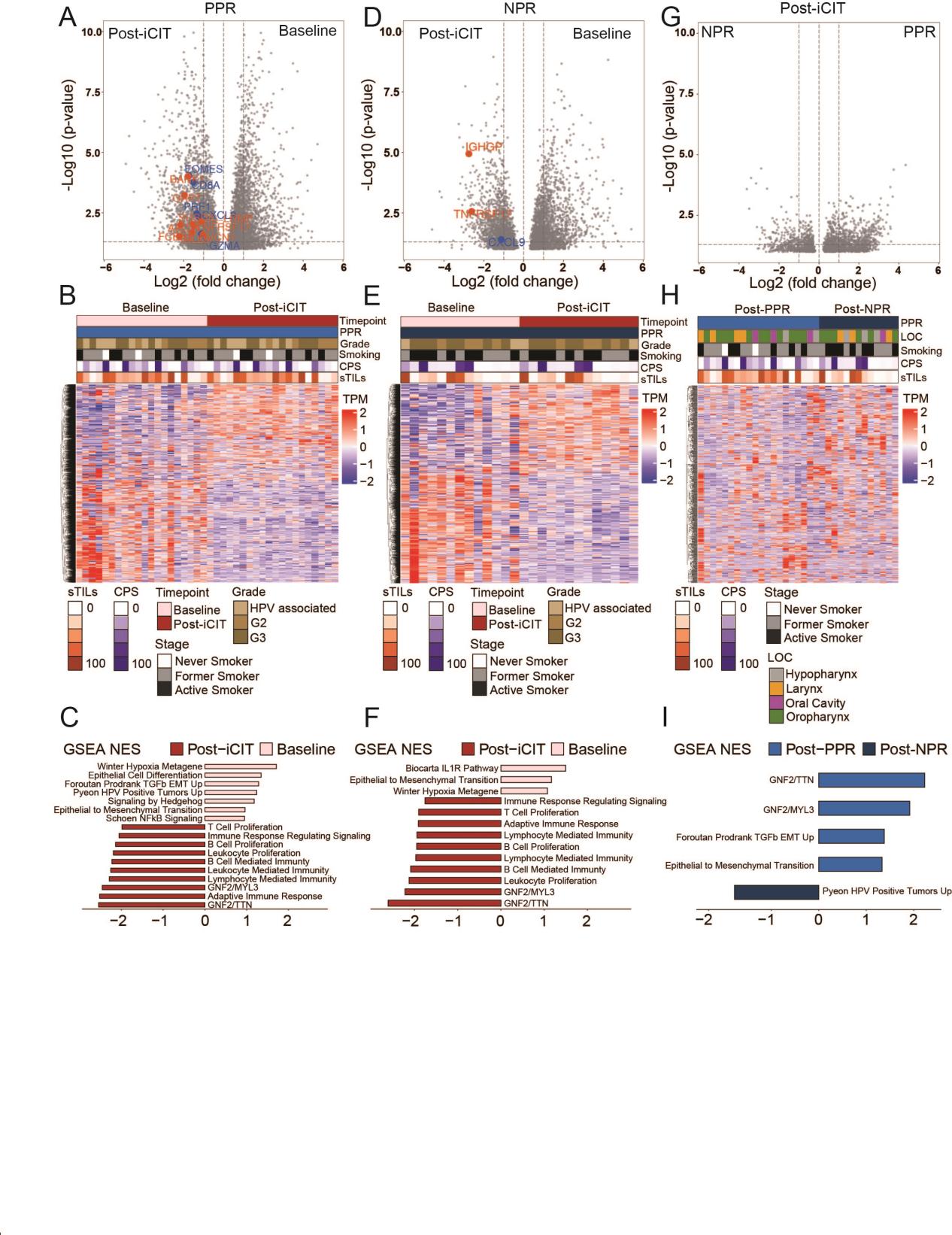


**Figure S2. Transcriptomic dynamics in pathological response patterns in locally advanced HNSCC patients with RD after iCIT.**

(A)Volcano plot depicting differentially expressed genes (FDR p<0.05; absolute logFC ≥1) between iCIT treated HNSCC patients with PPR at baseline versus post-iCIT. Hallmark B cell genes and T-effector genes are represented in darkgreen and darkred, respectively; (B) Hierarchical clustering of the timepoints identified from transcriptome analyses in PPR patients; (C) GSEA enrichment analysis for selected gene lists of differentially expressed genes (DEGs) in two groups (Baseline vs. post-iCIT) of PPR patients; (D) Volcano plot depicting differentially expressed genes (FDR p<0.05; absolute logFC ≥1) between iCIT treated HNSCC patients with NPR at baseline versus post-iCIT. Hallmark B cell genes and T-effector genes are represented in drakgreen and drakred, respectively; (E) Hierarchical clustering of the timepoints identified from transcriptome analyses in NPR patients; (F) GSEA enrichment analysis for selected gene lists of differentially expressed genes (DEGs) in two groups (Baseline vs. post-iCIT) of NPR patients; (G) Volcano plot depicting differentially expressed genes (FDR p<0.05; absolute logFC ≥1) between iCIT treated HNSCC patients with PPR versus NPR at post-iCIT. Hallmark B cell genes and T-effector genes are represented in drakgreen and drakred, respectively; (H) Hierarchical clustering of the PPR and NPR patterns identified from transcriptome analyses at post-iCIT; (I) GSEA enrichment analysis for selected gene lists of differentially expressed genes (DEGs) in two groups (PPR vs. NPR) at post-iCIT.


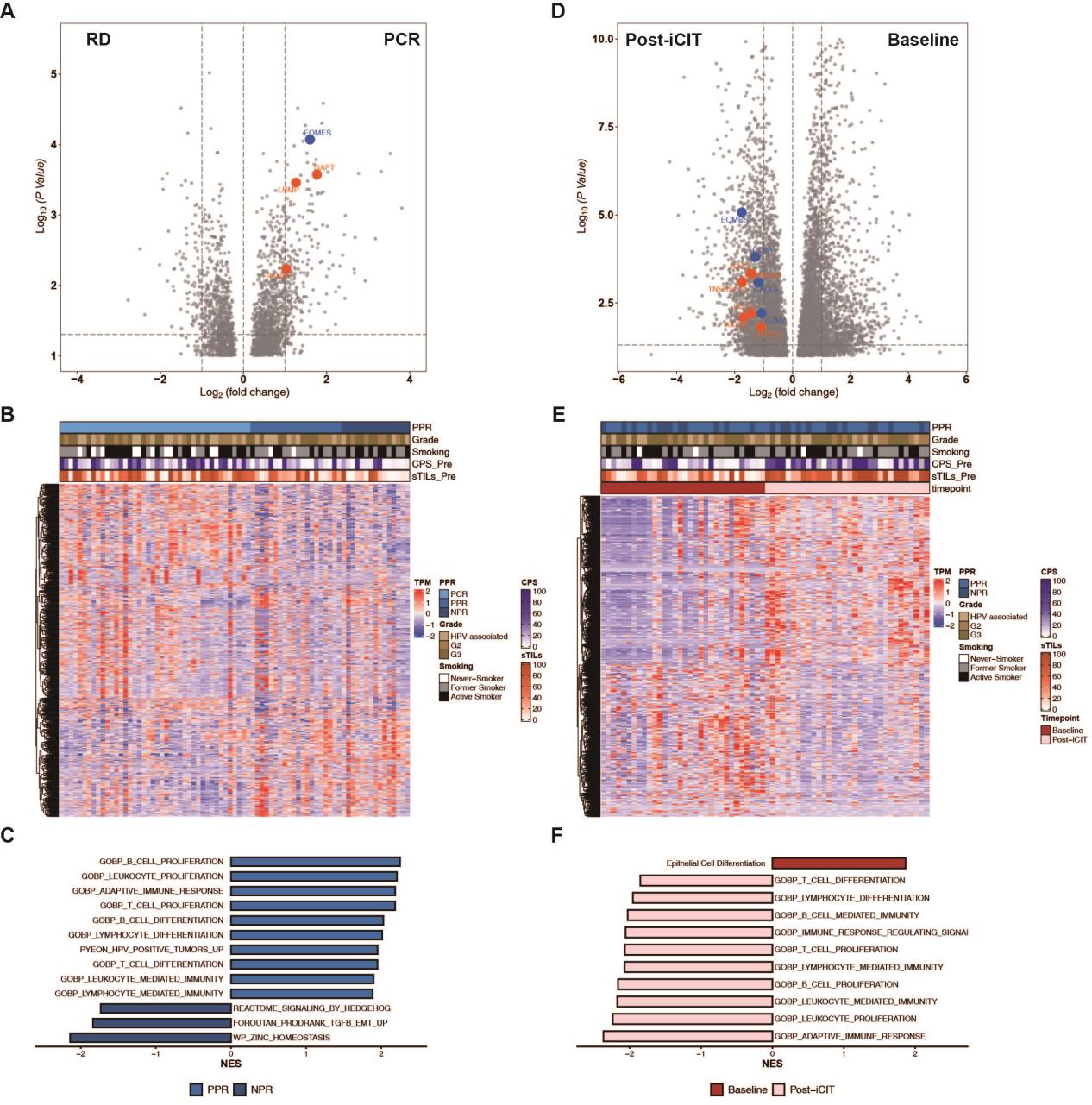


**Figure S3. Transcriptomic dynamics in pathological response patterns with tumor purity in patients.**

(A) Volcano plot depicting differentially expressed genes (FDR p<0.05; absolute logFC ≥1) between iCIT treated HNSCC patients with pCR (n=35) versus RD (n = 33) at baseline. Hallmark B cell genes and T-effector genes are represented in green and red, respectively; (B) Hierarchical clustering of the three pathological response patterns identified from transcriptome analyses; (C) GSEA enrichment analysis for selected gene lists of differentially expressed genes (DEGs) in two groups (pCR vs. RD); (D) Volcano plot depicting differentially expressed genes (FDR p<0.05; absolute logFC ≥1) between iCIT treated HNSCC patients with RD at baseline (n=33) versus at post-iCIT (n=33). Hallmark B cell genes and T-effector genes are represented in green and red, respectively; (E) Hierarchical clustering of the timepoints identified from transcriptome analyses; (F) GSEA enrichment analysis for selected gene lists of differentially expressed genes (DEGs) in two groups (Baseline vs. post-iCIT).


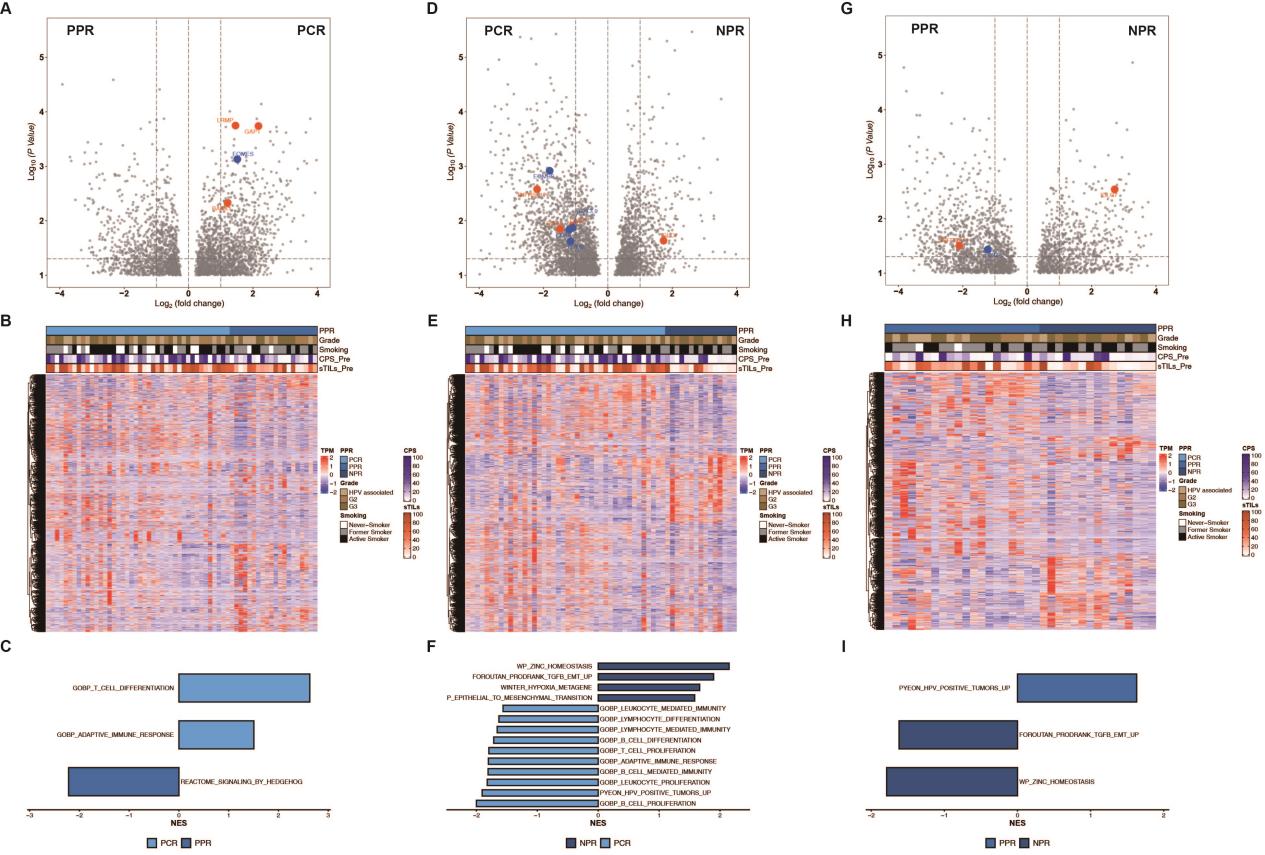


**Figure S4. Transcriptomic dynamics in pathological response patterns with tumor purity in patients at baseline.**

(A) Volcano plot depicting differentially expressed genes (FDR p<0.05; absolute logFC ≥1) between iCIT treated HNSCC patients with pCR versus PPR at baseline. Hallmark B cell genes and T-effector genes are represented in darkgreen and darkred, respectively; (B) Hierarchical clustering of the PPR and NPR patterns identified from transcriptome analyses; (C) GSEA enrichment analysis for selected gene lists of differentially expressed genes (DEGs) in two groups (pCR vs. PPR); (D) Volcano plot depicting differentially expressed genes (FDR p<0.05; absolute logFC ≥1) between iCIT treated HNSCC patients with pCR versus NPR at baseline. Hallmark B cell genes and T-effector genes are represented in drakgreen and drakred, respectively; (E) Hierarchical clustering of the pCR and NPR patterns identified from transcriptome analyses; (F) GSEA enrichment analysis for selected gene lists of differentially expressed genes (DEGs) in two groups (pCR vs. NPR); (G) Volcano plot depicting differentially expressed genes (FDR p<0.05; absolute logFC ≥1) between iCIT treated HNSCC patients with PPR versus NPR at baseline. Hallmark B cell genes and T-effector genes are represented in drakgreen and drakred, respectively; (H) Hierarchical clustering of the PPR and NPR patterns identified from transcriptome analyses.


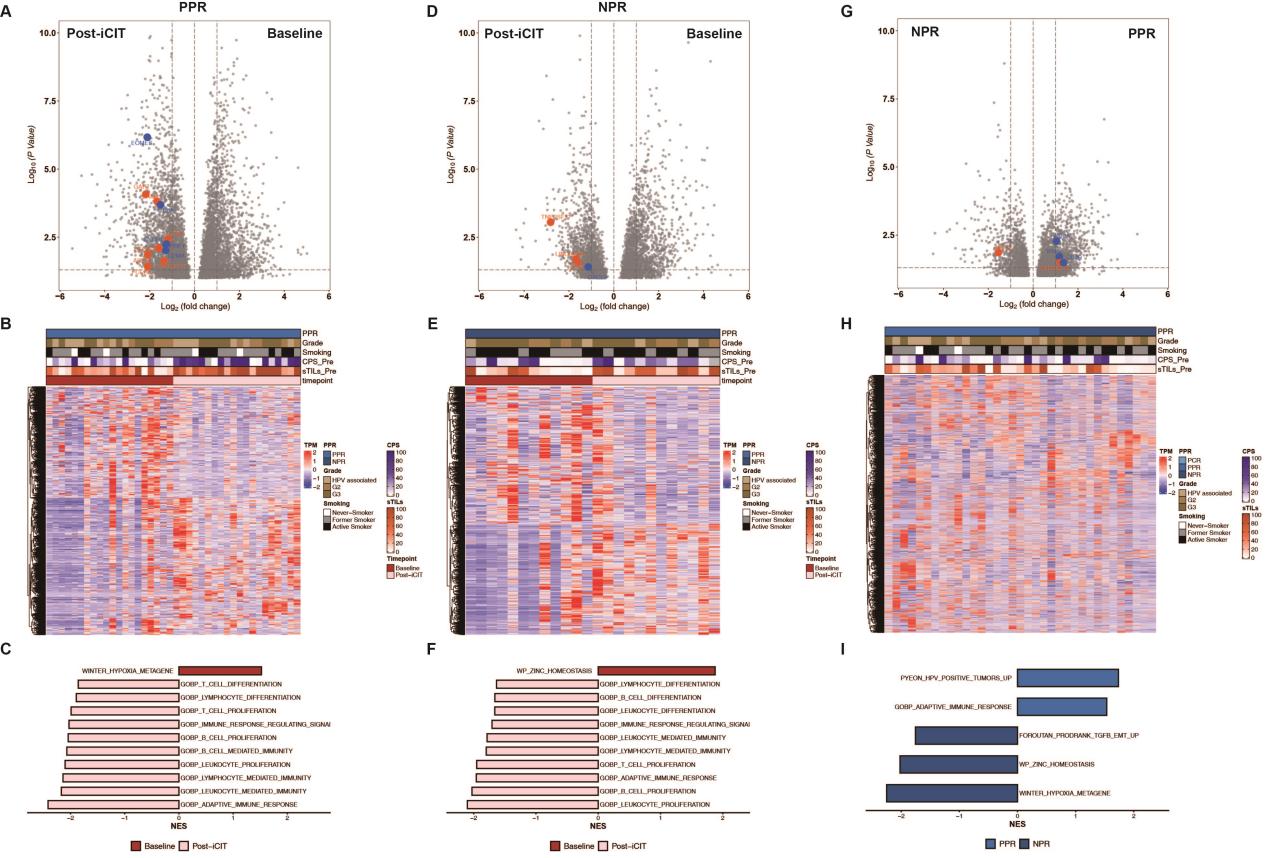


**Figure S5. Transcriptomic dynamics in pathological response patterns with tumor purity in patients with RD after iCIT.**

(A) Volcano plot depicting differentially expressed genes (FDR p<0.05; absolute logFC ≥1) between iCIT treated HNSCC patients with PPR at baseline versus post-iCIT. Hallmark B cell genes and T-effector genes are represented in darkgreen and darkred, respectively; (B) Hierarchical clustering of the timepoints identified from transcriptome analyses in PPR patients; (C) GSEA enrichment analysis for selected gene lists of differentially expressed genes (DEGs) in two groups (Baseline vs. post-iCIT) of PPR patients; (D) Volcano plot depicting differentially expressed genes (FDR p<0.05; absolute logFC ≥1) between iCIT treated HNSCC patients with NPR at baseline versus post-iCIT. Hallmark B cell genes and T-effector genes are represented in drakgreen and drakred, respectively; (E) Hierarchical clustering of the timepoints identified from transcriptome analyses in NPR patients; (F) GSEA enrichment analysis for selected gene lists of differentially expressed genes (DEGs) in two groups (Baseline vs. post-iCIT) of NPR patients; (G) Volcano plot depicting differentially expressed genes (FDR p<0.05; absolute logFC ≥1) between iCIT treated HNSCC patients with PPR versus NPR at post-iCIT. Hallmark B cell genes and T-effector genes are represented in drakgreen and drakred, respectively; (H) Hierarchical clustering of the PPR and NPR patterns identified from transcriptome analyses at post-iCIT; (I) GSEA enrichment analysis for selected gene lists of differentially expressed genes (DEGs) in two groups (PPR vs. NPR) at post-iCIT.

**
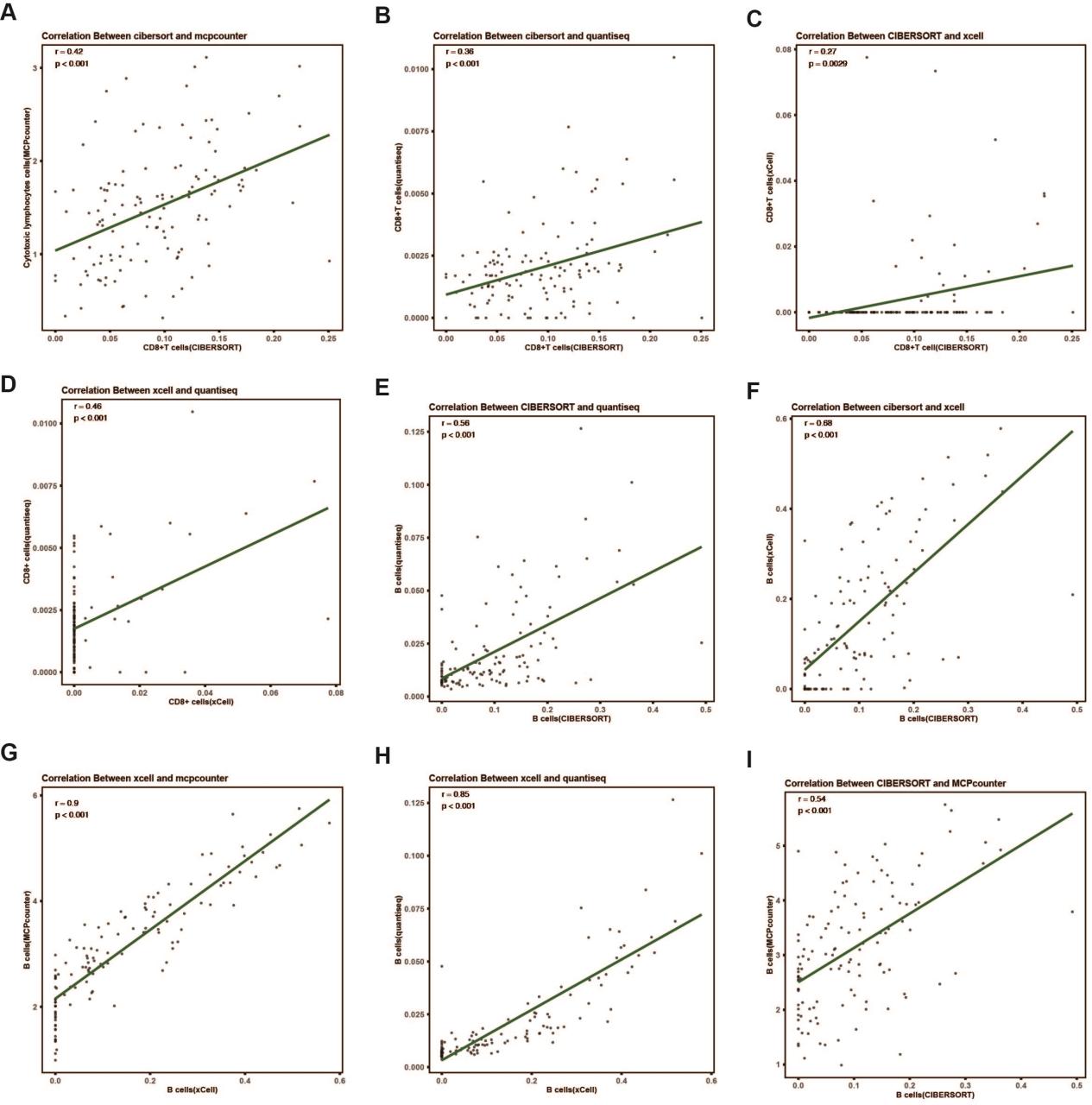
Figure S6. Correlation of CD8+ T cell and B cell between transcriptomic estimate.**

(A) CD8+ T cell correlation between cibersort method and mcpcounter method, R = 0.42, p < 0.001; (B) CD8+ T cell correlation between cibersort method and quantiseq method, R = 0.36, p < 0.001; (C) CD8+ T cell correlation between cibersort method and xCell method, R = 0.27, p = 0.0029; (D) CD8+ T cell correlation between quantiseq method and xCell method, R = 0.46, p < 0.001; (E) B cell correlation between cibersort method and quantiseq method, R = 0.56, p = 0.002; (F) B cell correlation between cibersort method and xCell method, R = 0.68, p < 0.001; (G) B cell correlation between mcpcounter method and xCell method, R = 0.9, p < 0.001; (H) B cell correlation between quantiseq method and xCell method, R = 0.85, p < 0.001; (I) B cell correlation between cibersort method and mcpcounter method, R = 0.54, p < 0.001.


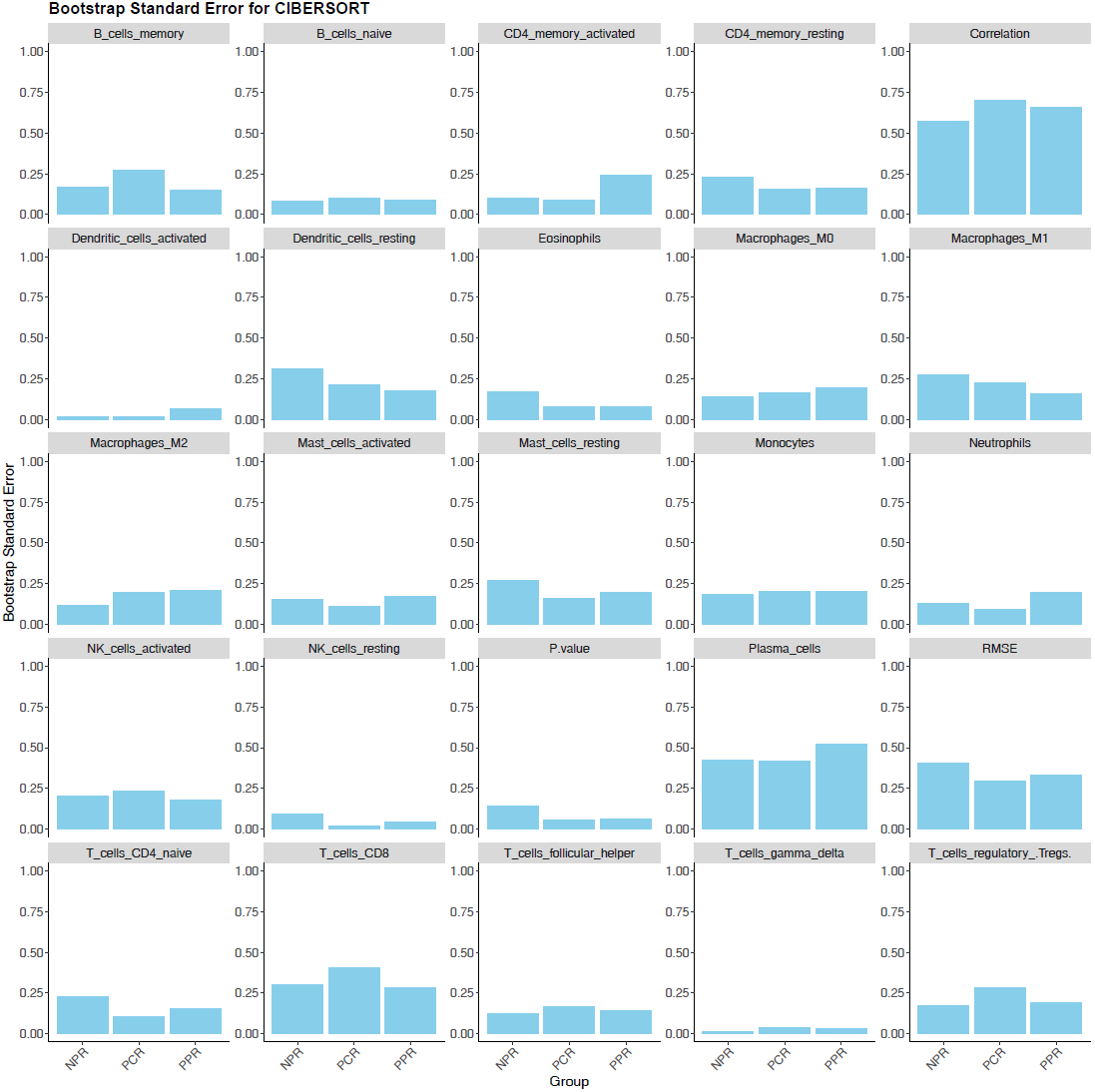


**Figure S7. Bootstrap analysis for CIBERSORT.**

Performed 1000 rounds of bootstrap resampling for each group and calculated the standard error for each cell type. The goal was to observe whether there were significant differences between the PCR, PPR, and NPR groups. If no significant differences were observed between the three groups, it would suggest good consistency in the results.


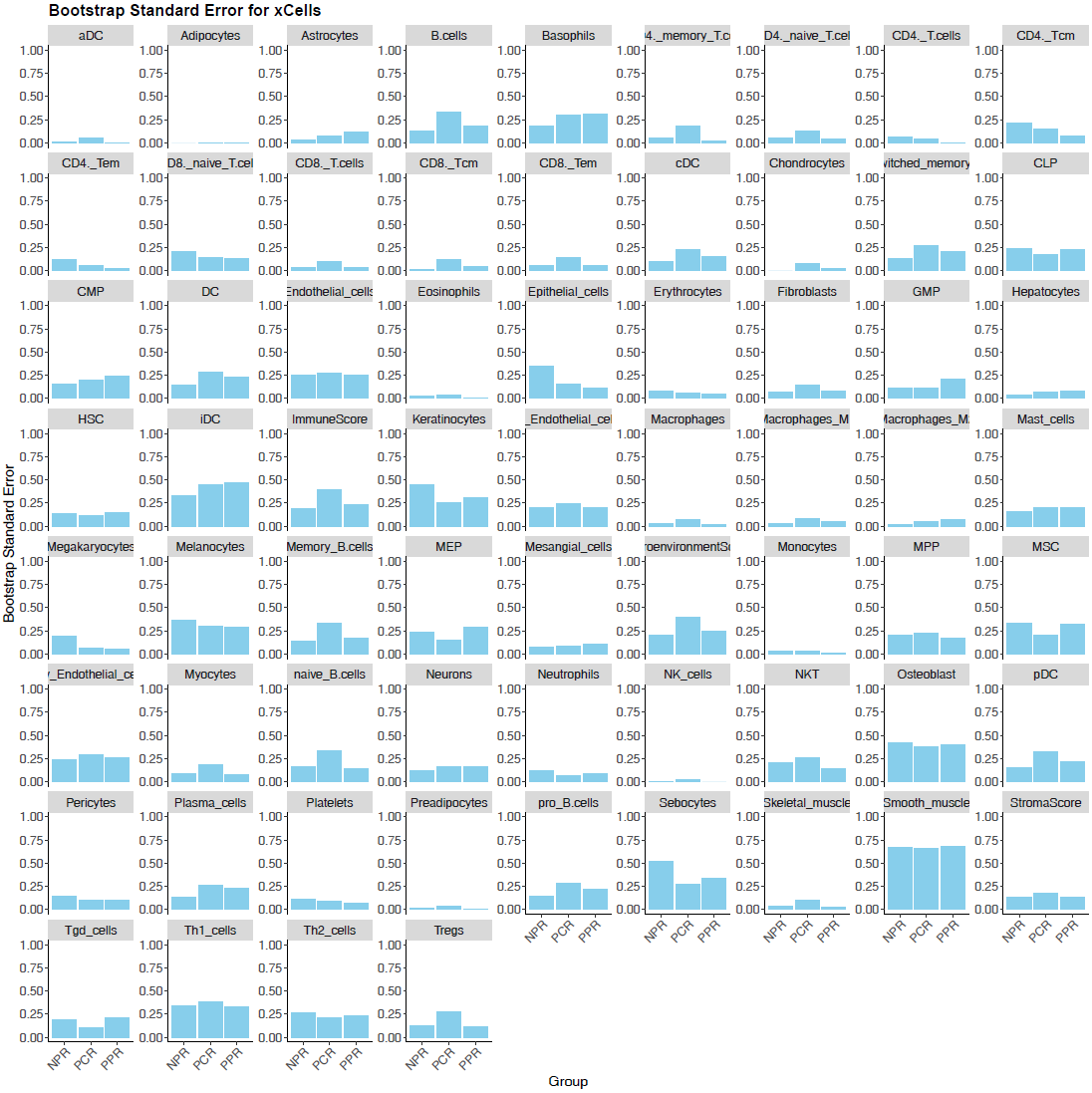


**Figure S8. Bootstrap analysis for xCells.**

Performed 1000 rounds of bootstrap resampling for each group and calculated the standard error for each cell type. The goal was to observe whether there were significant differences between the PCR, PPR, and NPR groups. If no significant differences were observed between the three groups, it would suggest good consistency in the results.


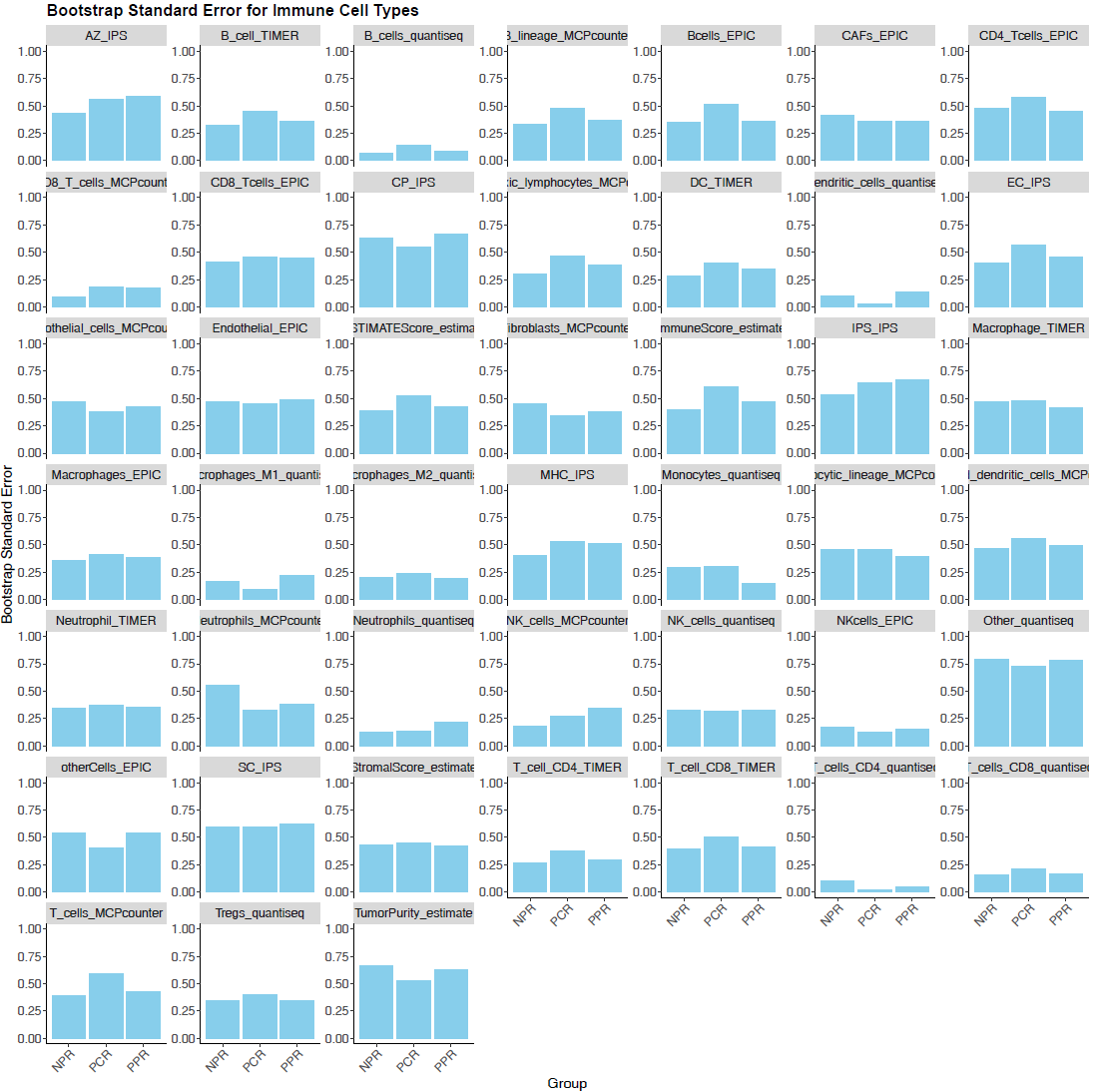


**Figure S9. Bootstrap analysis for other methods.**

Performed 1000 rounds of bootstrap resampling for each group and calculated the standard error for each cell type. The goal was to observe whether there were significant differences between the PCR, PPR, and NPR groups. If no significant differences were observed between the three groups, it would suggest good consistency in the results.


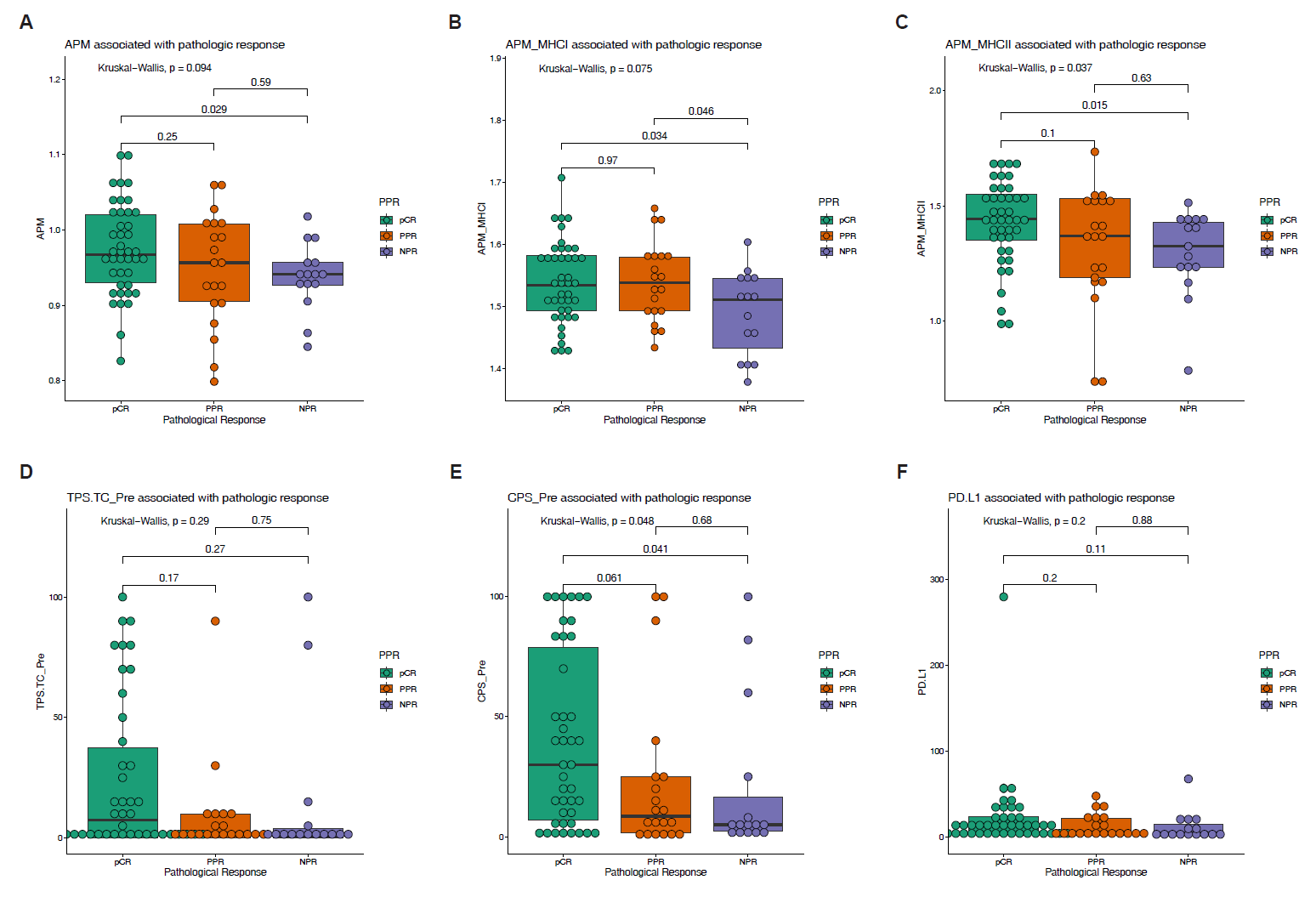


**Figure S10. Immune-checkpoint activity and antigen processing and presentation machinery associated with pathologic response patterns.**

(A) Boxplot of antigen processing and presentation machinery (APM) for samples from the three pathologic response groups. APM of samples from patient with pCR, PPR and NPR were compared using Wilcoxon rank-sum test; (B) Boxplot of MHCI related APM for samples from the three pathologic response groups. MHCI related APM of samples from patient with pCR, PPR and NPR were compared using Wilcoxon rank-sum test; (C) Boxplot of MHCII related APM for samples from the three pathologic response groups. MHCII related APM of samples from patient with pCR, PPR and NPR were compared using Wilcoxon rank-sum test; (D) Boxplot of TPS for samples from the three pathologic response groups. TPS of samples from patient with pCR, PPR and NPR were compared using Wilcoxon rank-sum test; (E) Boxplot of CPS for samples from the three pathologic response groups. CPS of samples from patient with pCR, PPR and NPR were compared using Wilcoxon rank-sum test; (F) Boxplot of PD-L1 for samples from the three pathologic response groups. PD-L1 of samples from patient with pCR, PPR and NPR were compared using Wilcoxon rank-sum test.


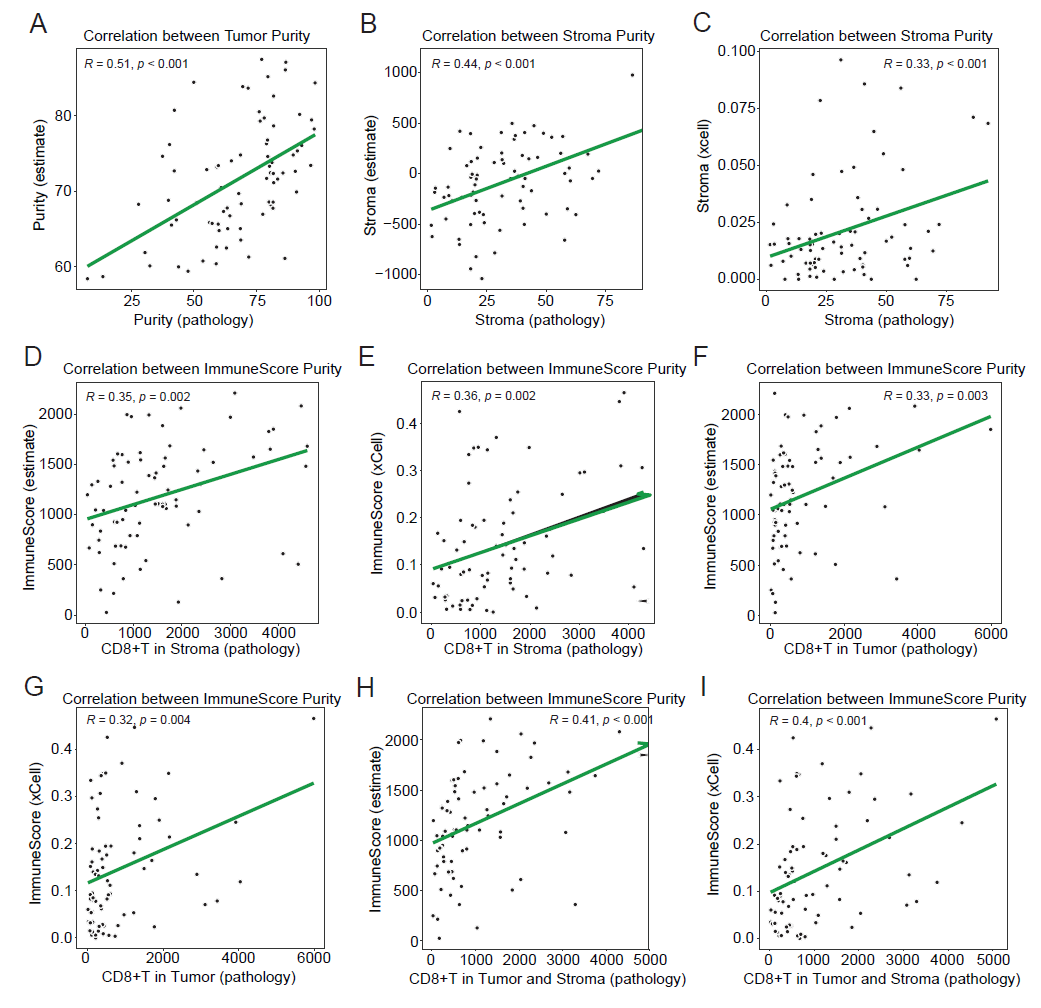


**Figure S11. Correlation of immune infiltration between pathology and transcriptomic estimate.**

(A) Correlation between Tumor Purity and Purity estimated by ***estimate*** method, R = 0.51, p < 0.001; (B) Correlation between Stroma Purity and Stroma estimated by ***estimate*** method, R = 0.44, p < 0.001; (C) Correlation between Stroma Purity and Stroma estimated by ***xCell*** method, R = 0.33, p < 0.001; (D) Correlation between CD8^+^T in Stroma and ImmuneScore estimated by ***estimate*** method, R = 0.35, p = 0.002; (E) Correlation between CD8^+^T in Stroma and ImmuneScore estimated by ***xCell*** method, R = 0.36, p = 0.002; (F Correlation between Tumor Purity and ImmuneScore estimated by ***estimate*** method, R = 0.33, p = 0.003; (G) Correlation between CD8+T in Tumor and ImmuneScore estimated by ***xCell*** method, R = 0.32, p = 0.004; (H) Correlation between **CD8+T in Tumor and Stroma** and ImmuneScore estimated by ***estimate*** method, R = 0.41, p < 0.001; (I) Correlation between **CD8+T in Tumor and Stroma** and ImmuneScore estimated by ***xCell*** method, R = 0.4, p < 0.001.


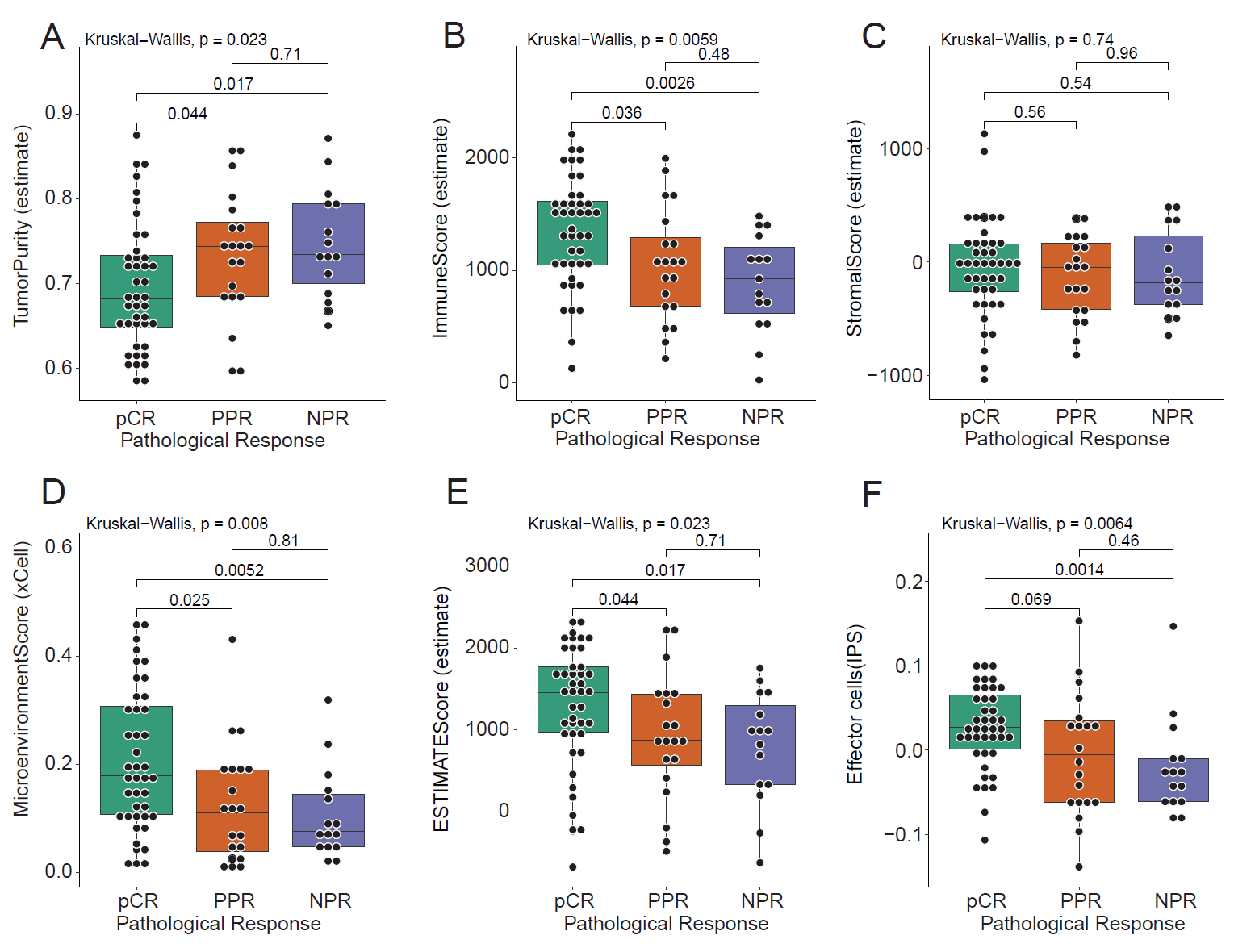


**Figure S12. Transcriptomic estimate characteristics associated with pathologic response patterns.**

1. Boxplot of TumorPurity for samples from the three pathologic response groups. TumorPurity of samples from patient with pCR, PPR and NPR were compared using Wilcoxon rank-sum test; (B) Boxplot of ImmuneScore for samples from the three pathologic response groups. ImmuneScore of samples from patient with pCR, PPR and NPR were compared using Wilcoxon rank-sum test; (C) Boxplot of StromalScore for samples from the three pathologic response groups. StromalScore of samples from patient with pCR, PPR and NPR were compared using Wilcoxon rank-sum test; (D) Boxplot of MicroenvironmentScore for samples from the three pathologic response groups. MicroenvironmentScore of samples from patient with pCR, PPR and NPR were compared using Wilcoxon rank-sum test; (E) Boxplot of ESTIMATEScore for samples from the three pathologic response groups. ESTIMATEScore of samples from patient with pCR, PPR and NPR were compared using Wilcoxon rank-sum test; (F) Boxplot of EC_IPS for samples from the three pathologic response groups. EC_IPS of samples from patient with pCR, PPR and NPR were compared using Wilcoxon rank-sum test.


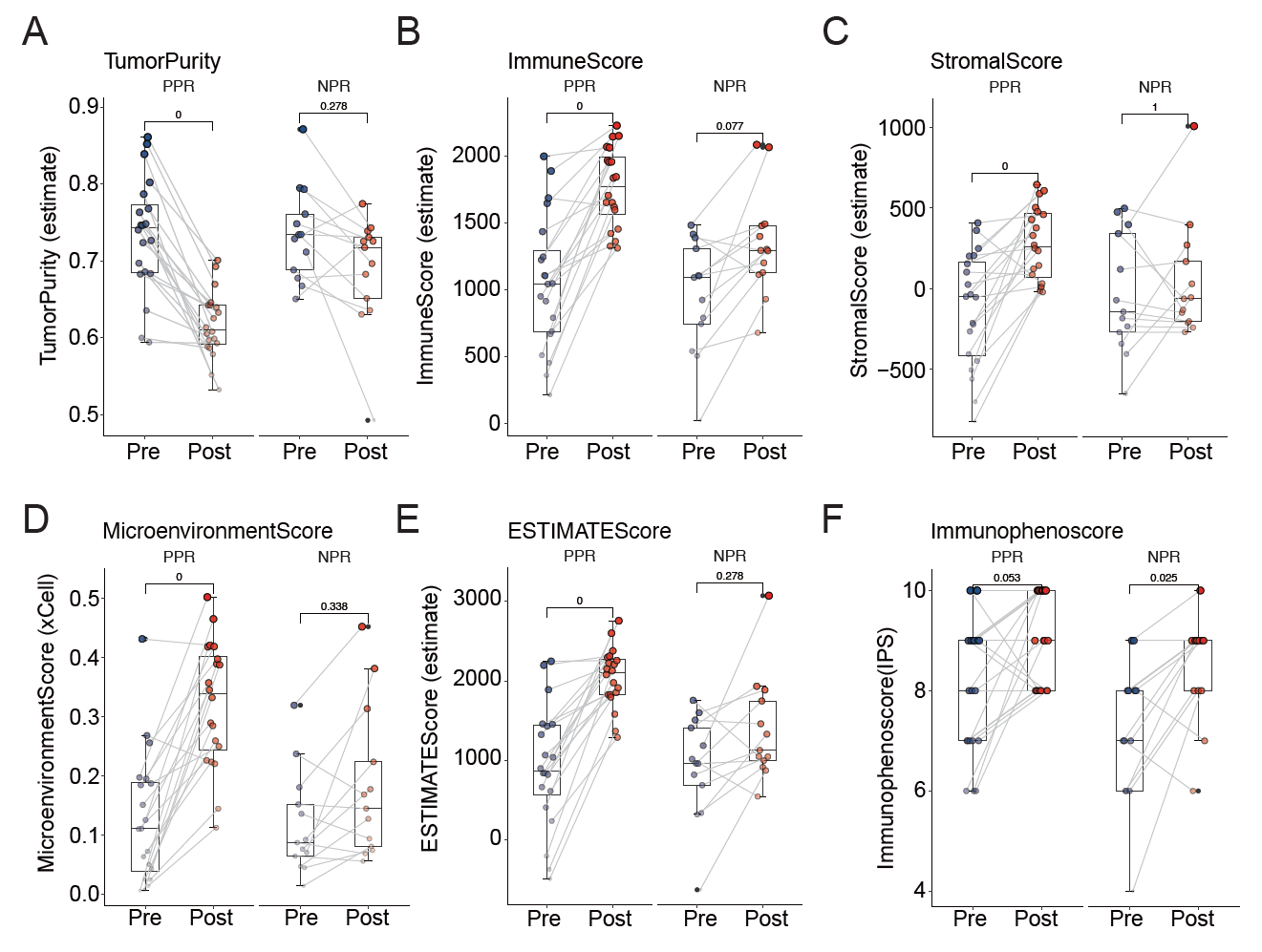


**Figure S13. Transcriptomic estimate characteristics dynamic change of response patterns between pre- and post-treatment.**

(A) Expression levels of TumorPurity in pre- and post-treatment samples of PPR and NPR group of patients. Statistical comparisons were performed using paired Wilcoxon signed-rank test; (B) Expression levels of ImmuneScore in pre- and post-treatment samples of PPR and NPR group of patients. Statistical comparisons were performed using paired Wilcoxon signed-rank test; (C) Expression levels of StromalScore in pre- and post-treatment samples of PPR and NPR group of patients. Statistical comparisons were performed using paired Wilcoxon signed-rank test; (D) Expression levels of MicroenvironmentScore in pre- and post-treatment samples of PPR and NPR group of patients. Statistical comparisons were performed using paired Wilcoxon signed-rank test; (E) Expression levels of ESTIMATEScore in pre- and post-treatment samples of PPR and NPR group of patients. Statistical comparisons were performed using paired Wilcoxon signed-rank test; (F) Expression levels of IPS_IPS in pre- and post-treatment samples of PPR and NPR group of patients. Statistical comparisons were performed using paired Wilcoxon signed-rank test.


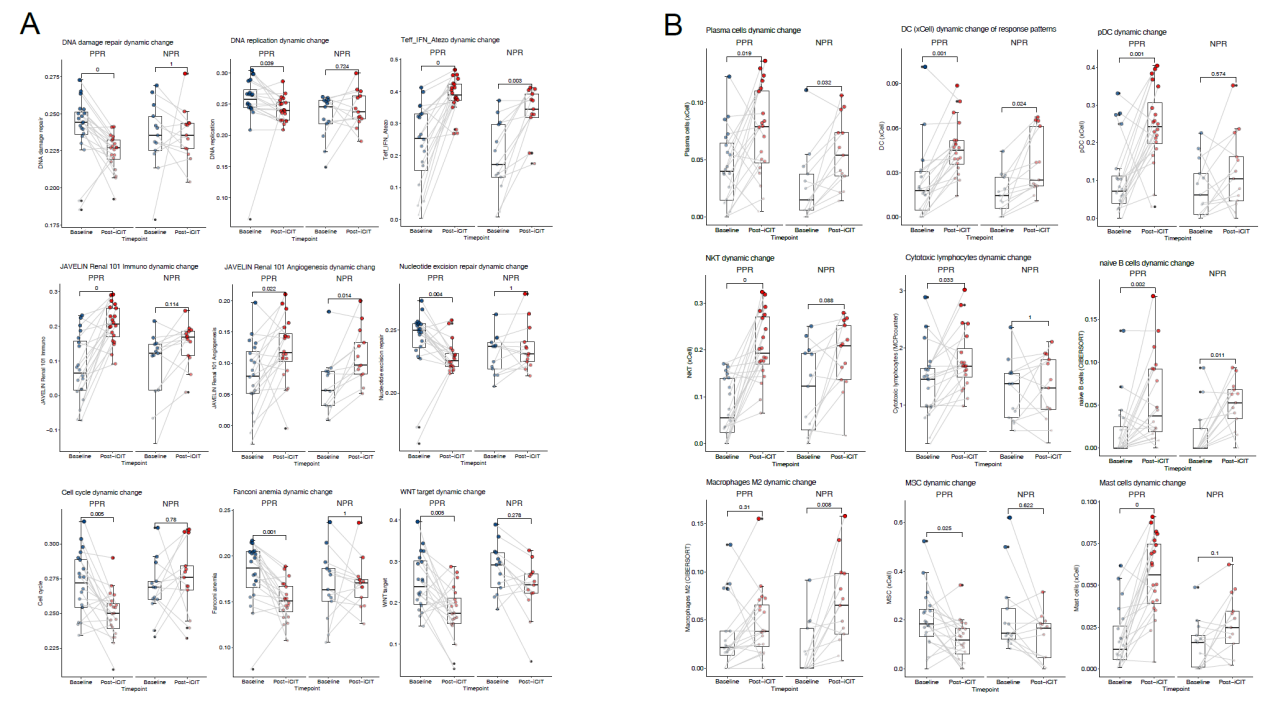


**Figure S14. Transcriptional signature and TIME dynamic change of response patterns between pre- and post-treatment.**

1. Expression levels of transcriptional signatures in pre- and post-treatment samples of PPR (n=20) and NPR (n=13) group of patients. Statistical comparisons were performed using paired Wilcoxon signed-rank test; (B) Abundances of TIME subtypes in pre- and post-treatment samples of PPR (n=20) and NPR (n=13) group of patients. Statistical comparisons were performed using paired Wilcoxon signed-rank test.


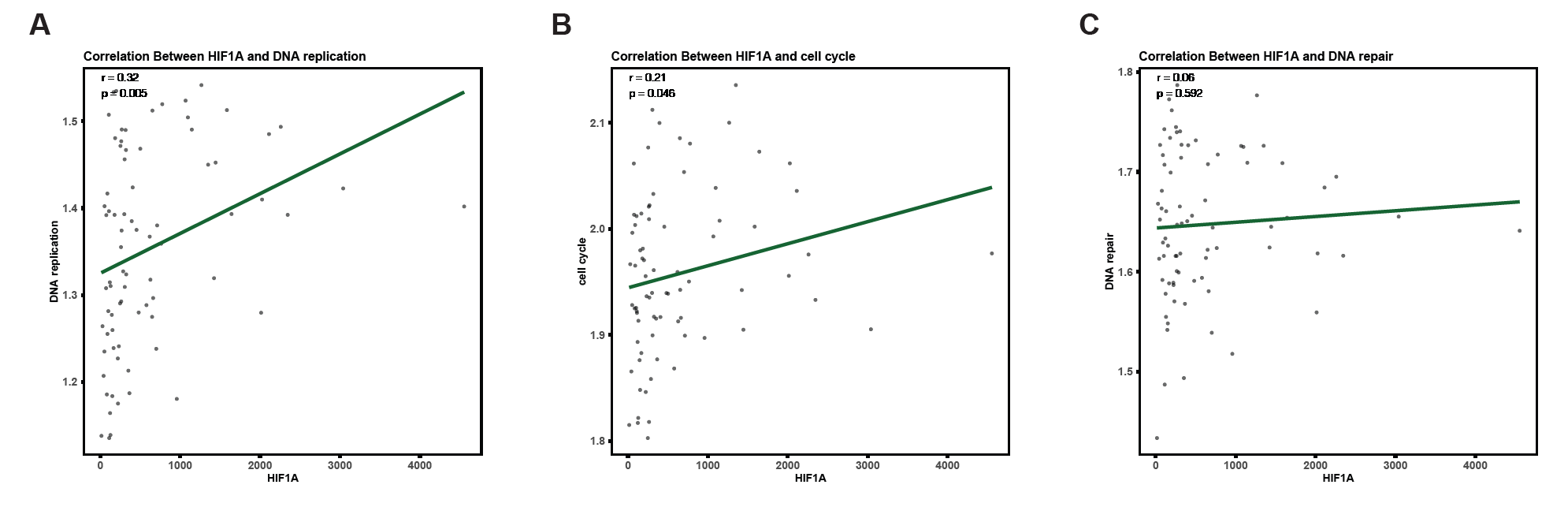


**Figure S15. Correlation analysis between HIF1A and relevant pathway.**

(A) Correlation between expression of HIF1A and DNA replication, R = 0.32, p =0.05; (B) Correlation between expression of HIF1A and cell cycle, R = 0.21, p = 0.046; (C) Correlation between expression of HIF1A and DNA damage repair, R = 0.06, p = 0.592.


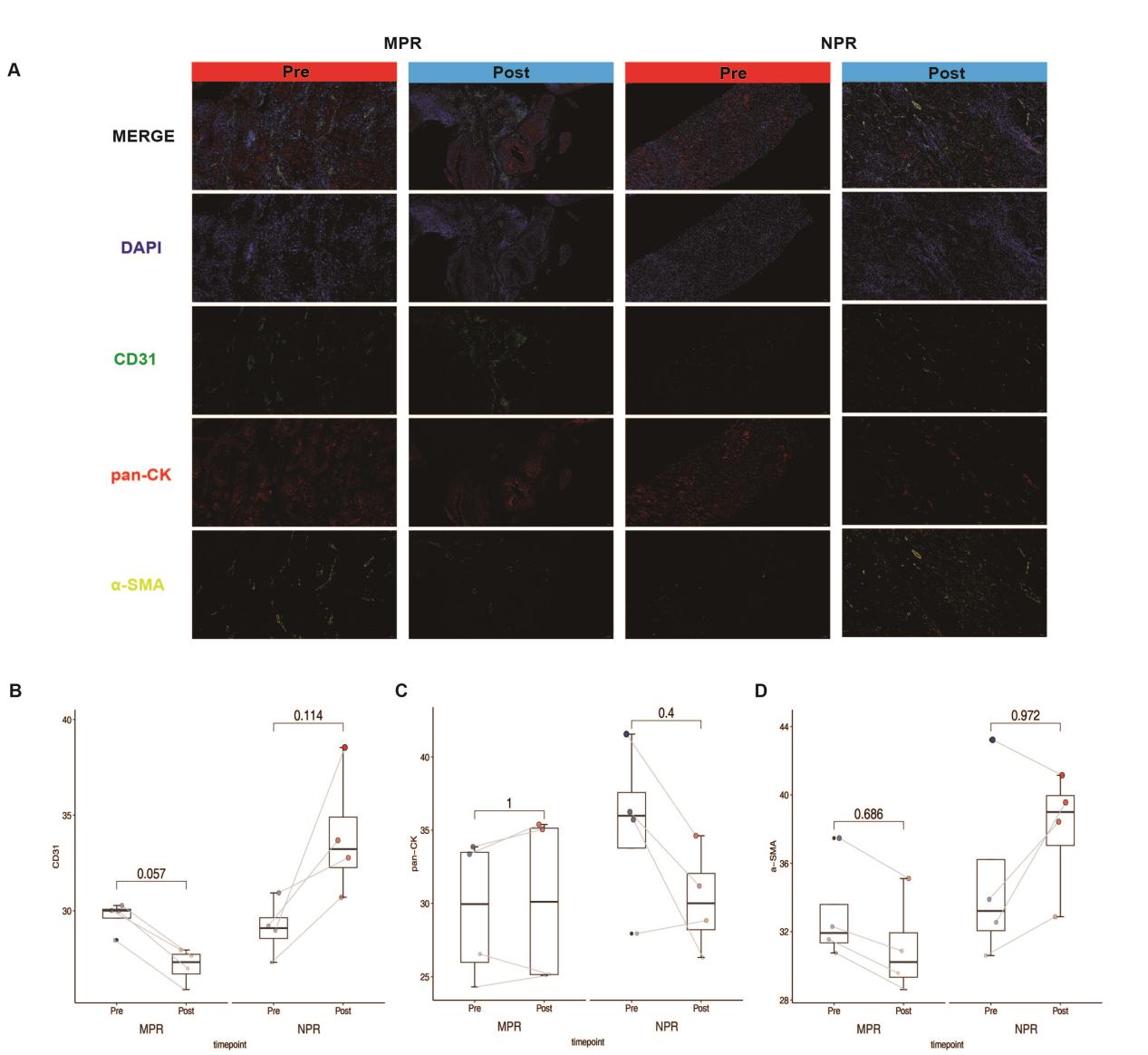


**Figure S16. Correlation of epithelial-mesenchymal transition between pre- and post-treatment.**

(A) The confocal microscopy images showed the co-expression of CD31, pan-CK, α-SMA in each pathological response pattern and treatment stage by immunofluorescence analysis. DAPI: blue, α-SMA: yellow, CD31: green, pan-CK: red. Scale bar, 100 μm. Boxplots showing the changes in expression of CD31 (B), pan-CK (C), and α-SMA (D) from pre-treatment to post-treatment in the MPR and NPR groups. Paired statistical analysis is performed, with p-values shown for each comparison.


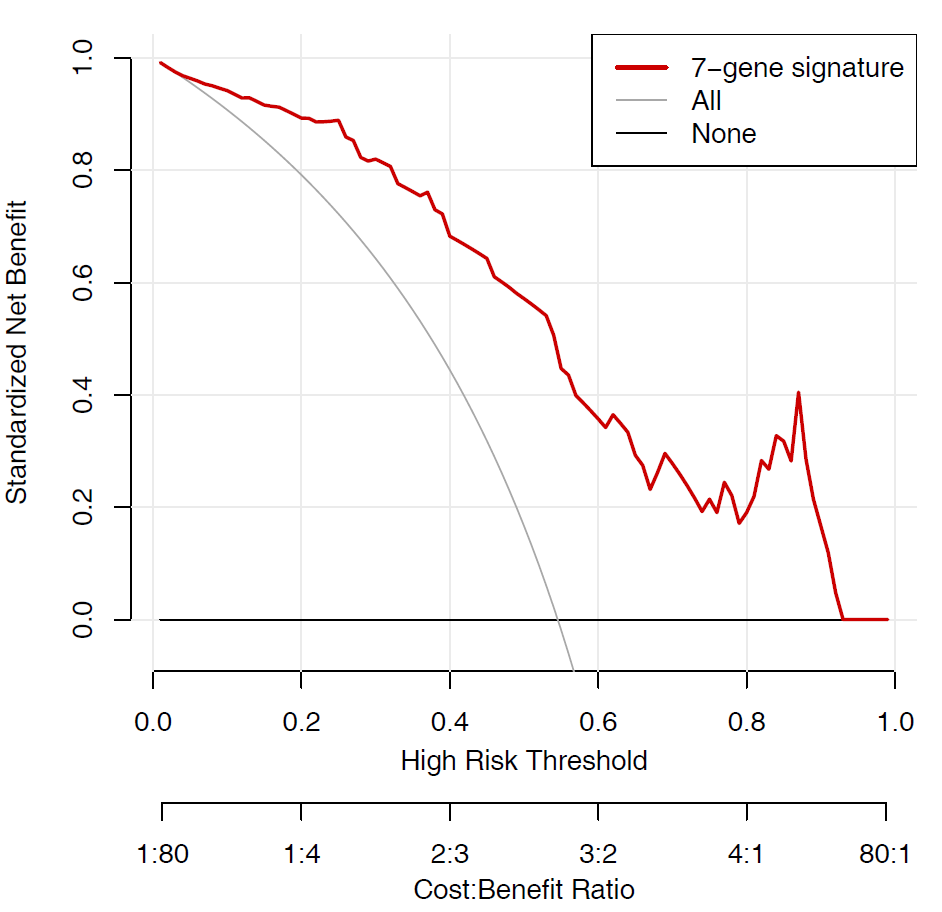


**Figure S17. Decision Curve Analysis for CheckRad-7-gene signature.**

The DCA demonstrates the net benefit of the 7-gene signature has a higher net benefit compared to the "treat all" and "treat none" strategies, over the entire range of reasonable threshold probabilities.


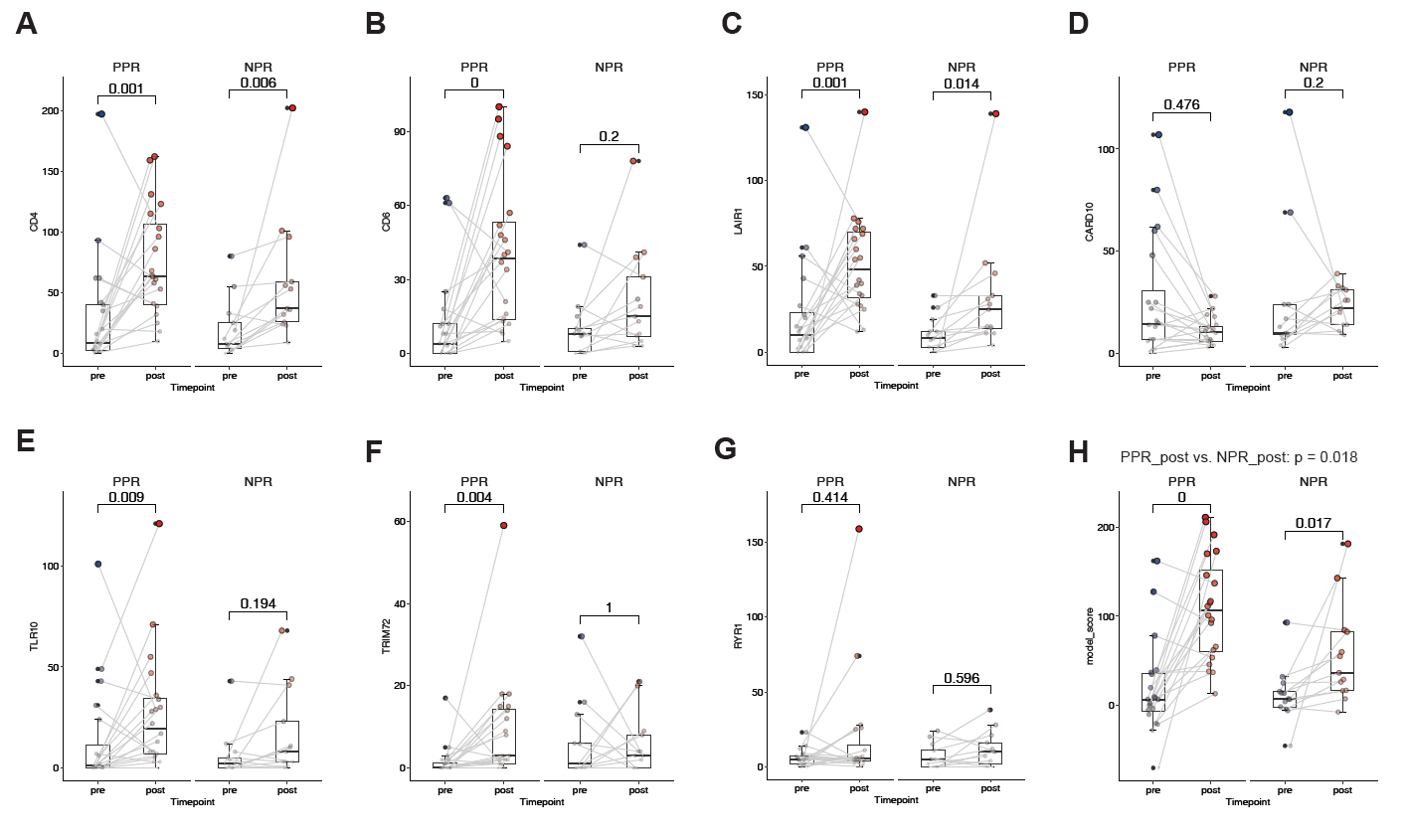


**Figure S18. CheckRad-7-gene signature dynamic change of response patterns between pre- and post-treatment.**

(A-G) Expression levels of CheckRad-7-gene signatures in pre- and post-treatment samples of PPR and NPR group of patients. Statistical comparisons were performed using paired Wilcoxon signed-rank test; (H) CheckRad-7-gene signature score in pre- and post-treatment samples of PPR and NPR group of patients. Statistical comparisons were performed using paired Wilcoxon signed-rank test.


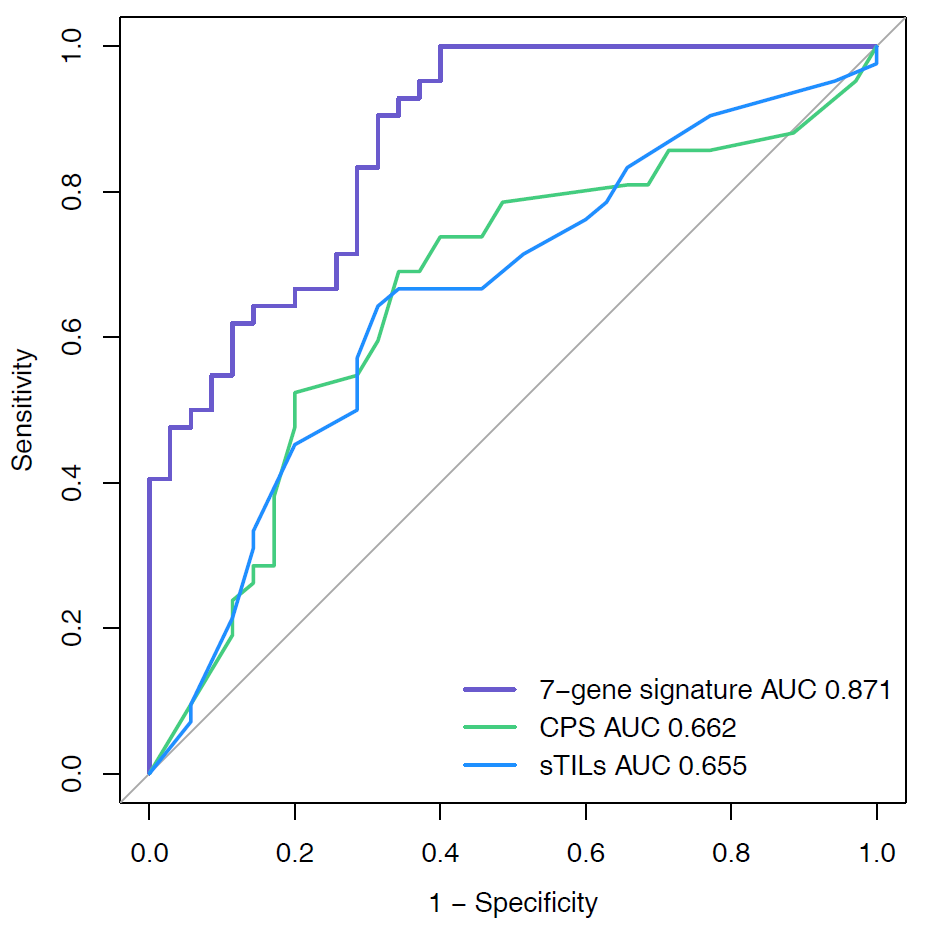


**Figure S19. ROC curves for the 7-gene signature, CPS, and sTILs.**

Receiver operating characteristic (ROC) curves comparing the predictive performance of the 7-gene signature, CPS, and sTILs. The curves represent sensitivity versus 1-specificity at various threshold values.
